# GABA_A_ receptor subunit deregulation in the hippocampus of human foetuses with Down syndrome

**DOI:** 10.1007/s00429-017-1563-3

**Published:** 2017-11-22

**Authors:** Ivan Milenkovic, Tamara Stojanovic, Eleonora Aronica, Livia Fülöp, Zsolt Bozsó, Zoltán Máté, Yuchio Yanagawa, Homa Adle-Biassette, Gert Lubec, Gábor Szabó, Tibor Harkany, Gábor G. Kovács, Erik Keimpema

**Affiliations:** 1 Department of Neurology, Medical University of Vienna, AKH 6A, Währinger Gürtel 18-20, 1097 Vienna, Austria; 2 Neurodegeneration Research Group, Institute of Neurology, Medical University of Vienna, Vienna, Austria; 3 Department of (Neuro) Pathology, Academic Medical Center and Swammerdam Institute for Life Sciences, Center for Neuroscience, University of Amsterdam, Amsterdam, The Netherlands; 4 Stichting Epilepsie Instellingen Nederland (SEIN), Amsterdam, The Netherlands; 5 Department of Medical Chemistry, University of Szeged, Szeged, Hungary; 6 Institute of Experimental Medicine, Hungarian Academy of Sciences, Szigony u. 43, 1083 Budapest, Hungary; 7 Department of Genetic and Behavioral Neuroscience, Gunma University School of Medicine, Maebashi, Japan; 8 Service d’Anatomie et Cytologie Pathologiques, Faculté de Médecine, Université Paris Diderot, Paris, France; 9 Paracelsus Medical University, Salzburg, Austria; 10 Center for Brain Research, Department of Molecular Neurosciences, Medical University of Vienna, Spitalgasse 4, 1090 Vienna, Austria; 11 Department of Neuroscience, Karolinska Institutet, Stockholm, Sweden

**Keywords:** Interneuron, Differentiation, Pyramidal cell, Cell migration, Neurodegeneration

## Abstract

The function, regulation and cellular distribution of GABA_A_ receptor subunits have been extensively documented in the adult rodent brain and are linked to numerous neurological disorders. However, there is a surprising lack of knowledge on the cellular (sub-) distribution of GABA_A_ receptor subunits and of their expressional regulation in developing healthy and diseased foetal human brains. To propose a role for GABA_A_ receptor subunits in neurodevelopmental disorders, we studied the developing hippocampus of normal and Down syndrome foetuses. Among the α1–3 and γ2 subunits probed, we find significantly altered expression profiles of the α1, α3 and γ2 subunits in developing Down syndrome hippocampi, with the α3 subunit being most affected. α3 subunits were selectively down-regulated in all hippocampal subfields and developmental periods tested in Down syndrome foetuses, presenting a developmental mismatch by their adult-like distribution in early foetal development. We hypothesized that increased levels of the amyloid precursor protein (APP), and particularly its neurotoxic β-amyloid (1–42) fragment, could disrupt α3 gene expression, likely by facilitating premature neuronal differentiation. Indeed, we find increased APP content in the hippocampi of the Down foetuses. In a corresponding cellular model, soluble β-amyloid (1–42) administered to cultured SH-SY5Y neuroblastoma cells, augmented by retinoic acid-induced differentiation towards a neuronal phenotype, displayed a reduction in α3 subunit levels. In sum, this study charts a comprehensive regional and subcellular map of key GABA_A_ receptor subunits in identified neuronal populations in the hippocampus of healthy and Down syndrome foetuses and associates increased β-amyloid load with discordant down-regulation of α3 subunits.

**Electronic supplementary material:**

The online version of this article (10.1007/s00429-017-1563-3) contains supplementary material, which is available to authorized users.

## Introduction

γ-Aminobutyric acid (GABA) is the main inhibitory neurotransmitter in the adult brain, which signals through GABA_A_ and GABA_B_ receptors (GABA_A/B_-Rs) (Johnston et al. 1978; Wilkin et al. 1981). GABA_A_-Rs are ligand-gated ion channels organized by pentameric assembly from a panoply of nineteen subunits (Sieghart 1995). Although theoretically, many arrangements are possible to form a pentameric receptor, only a limited number of combinations seem to confer functionality in vivo (Olsen and Sieghart 2008). By virtue of their distinct subunit composition, GABA_A_-Rs show substantial diversity in their biophysical and pharmacological properties, as well as distribution throughout the brain (Belelli et al. 2009; Eyre et al. 2012; Hortnagl et al. 2013; Pirker et al. 2000; Ramerstorfer et al. 2011; Sieghart 1995; Sieghart et al. 2012; Varagic et al. 2013a, b). Their regional diversity in the adult brain allows GABA_A_-Rs to drive region- and cell-type-specific inhibition, underlying, e.g., sensory and motor processing, sleep-wakefulness, emotional control, learning, memory and cognition (Fritschy and Panzanelli 2014).

A pivotal role for GABA_A_-Rs during embryonic brain development has been proposed because of their gradual enrichment from early foetal development (Cobas et al. 1991; Fiszman et al. 1993; Stojanovic et al. 2016) to gate the GABA-mediated control of cell proliferation (Martinez-Cue et al. 2013), migration (Behar et al. 2000; Heck et al. 2007) and differentiation (Cuzon et al. 2006), particularly for cortical interneurons and pyramidal cells. Since impaired GABA signaling in development and adulthood is strongly correlated with pathological states associated with excess excitation, including epilepsy (Pavlov et al. 2011), anxiety and depression (Kalueff and Nutt 2007), a possible pathological contribution of altered GABA_A_-R-mediated signalling has been posited in neurodevelopmental disorders such as Rett syndrome (Blue et al. 1999; Yamashita et al. 1998), fragile X syndrome (D’Hulst et al. 2006), Prader-Willi/Angelman syndrome (Braat and Kooy 2015) and Down syndrome (Braudeau et al. 2011; Martinez-Cue et al. 2014; Potier et al. 2014).

Down syndrome (trisomy 21) is the most common congenital cause of mental retardation with an incidence of approximately 1 in 750 births (Gardiner et al. 2010; Lott and Dierssen 2010; Parker et al. 2010). Histological studies showed that disrupted proliferation and migration of neurons and glial cells in human foetal brains with Down syndrome is associated with reduced hippocampal and cortical volume, delamination and delayed myelination (Abraham et al. 2012; Contestabile et al. 2007; Golden and Hyman 1994; Guidi et al. 2008; Kanaumi et al. 2013; Larsen et al. 2008). Recent studies in mouse models of Down syndrome showed that excess inhibition through GABA_A_-Rs could underlie morphological deficits and the ensuing cognitive decline (Braudeau et al. 2011; Fernandez et al. 2007; Martinez-Cue et al. 2013). As such, RO4938581, a GABA_A_-R α5 subunit-specific negative allosteric modulator, rescued these learning and memory deficits (Martinez-Cue et al. 2013, 2014). However, if the developmentally altered GABA_A_-R subunit expression profiles could underlie phenotypic deficits of specific neuronal contingents in Down syndrome remain unknown.

Here we sought to investigate the distribution of α1, α2, α3 and γ2 GABA_A_-R subunits at the network, cellular and subcellular levels in the developing human hippocampal formation from healthy subjects and age-matched cases with Down syndrome. These particular subunits were chosen, since α2 and α3 are predominantly expressed during in-utero development in rodents with their substitution for α1 subunits timed for around birth (Fritschy et al. 1994). Together with their widespread pairing with the γ2 subunit (Hortnagl et al. 2013), we probed possible alterations to the spatiotemporal hippocampal distribution in human Down syndrome foetuses and dissected cell-type-specific enrichment in mouse hippocampi. We find that the spatiotemporal expression of the α3 subunit is especially disrupted in Down syndrome, seen as a premature down-regulation in the CA1–CA3 subfields and dentate gyrus, mimicking an adult-like phenotype at early developmental stages. Since β-amyloid deposits are correlated with the cognitive decline of the ageing brain (Rodrigue et al. 2009), and Down syndrome patients have elevated β-amyloid(1–42) levels in plasma (Obeid et al. 2016) and present early β-amyloid plaque formation (Motte and Williams 1989), we hypothesized that soluble β-amyloid(1–42), produced from an increased pool of amyloid precursor protein (APP) encoded on chromosome 21 (Korenberg et al. 1989) and linked to cellular growth responses (Freude et al. 2011; Wang et al. 2009), is responsible for the adult-like phenotype observed in late-gestational foetuses. Indeed, in our cell culture models, including human SH-SY5Y neuroblastoma cells, we find that soluble β-amyloid(1–42) promotes the progression of differentiating neuroblasts towards a neuron-like phenotype with coincident down-regulation of α3 subunits. This suggests that β-amyloid(1–42) might partake in imposing premature maturation events in the brains of Down syndrome subjects.

## Methods

### Human tissue collection and cataloguing

We collected 28 cases with diagnosed Down syndrome (by karyotyping) and 24 age-matched cases of normal brain development and adulthood at the Brain Bank of the Institute of Neurology, Medical University of Vienna, Austria (Nr. 1316/2012). Foetal brain tissue was obtained from spontaneous or medically induced abortions with their parameters published previously (Milenkovic et al. 2017). Only brains of foetuses [15–34 gestational weeks (GW)], infants (up to 6 months of the early postnatal period) and adults (15–62 years) whose cause of death was unrelated to other genetic disorders, head injury, neurological diseases or other known diseases (e.g. infections) were included. Exclusion criteria were other chromosome aberrations, major CNS malformations, as well as brains with post-mortem autolysis, severe hypoxic/ischemic encephalopathy, intraventricular haemorrhages, severe hydrocephalus, meningitis or ventriculitis (Supplementary Table 1). Biological periods were defined according to paediatric guidelines (Kanaumi et al. 2013): period 1 included early pregnancy period [14–16 gestational weeks (GW)], period 2 spanned the middle pregnancy period (16–28 GW), period 3 corresponded to the late pregnancy period (28 GW onwards, newborns and babies) and adults (15–62 years). Adult cases were used for photographic illustrations, but excluded from our statistical evaluation. Tissue was obtained and used according to the Declaration of Helsinki and compatible institutional guidelines (Kanaumi et al. 2013).

### Foetal tissue processing for immunohistochemistry

Foetal tissues were kept at 4° for 24–48 h due to local regulations before immersion fixation in formalin (10%). Tissues were kept in formalin on average for 34–37 days before embedding in paraffin. Formalin-fixed, paraffin-embedded tissue blocks containing the hippocampus and temporal and insular cortices were cut in the coronal plane at 3-µm thickness and mounted on gelatin pre-coated glass slides (Star Frost). Anatomical regions were delineated according to the atlas of human central nervous system development (Bayer and Altman 2005) on hematoxylin–eosin-stained specimens. The immunohistochemical staining methods used here were described previously (Alpar et al. 2014; Kanaumi et al. 2013). In brief, following deparaffinization and rehydration, sections were preincubated either in low pH EnVision™ FLEX antigen retrieval solution at 98 °C for 20 min (BD24 and α3) or with high pH (α2 and γ2), and subsequently manually stained overnight with antibody concentrations listed in Supplementary Table 3. DAKO’s EnVision detection kit was used to visualize the horseradish peroxidase/3,3′-diaminobenzidine (DAB) reaction with H_2_O_2_ substrate (0.01%; DAKO). Sections were counterstained with hematoxylin–eosin, dehydrated in ascending concentrations of ethanol, cleared with xylene and covered with Consil-Mount (Thermo Scientific). Sections were inspected on a Nikon Eclipse E400 microscope.

For mouse tissues, sections were blocked with 5% normal donkey serum (NDS; Jackson), 2% bovine serum albumin (BSA; Sigma) and 0.3% Triton X-100 (Sigma) in PBS. Next, sections were incubated with select combinations of primary antibodies (Supplementary Table 3) in 2% NDS, 0.1% BSA and 0.3% Triton X-100 in PBS at 4 °C for 72 h. Secondary antibodies were applied at a concentration of 1:300 at 22–24 °C for 2 h with 2% BSA in PBS. Sections were routinely counterstained with Hoechst 33,342 (Sigma), a nuclear marker, before being coverslipped with Entellan (in toluene; Merck).

### Animal tissue

Tissue collection from live animals conformed to the 2010/63/EU directive and was approved by the Austrian Ministry of Science and Research (66.009/0145-WF/II/3b/2014). Particular care was taken to minimize the number and suffering of experimental subjects. Adult mice [C57BL/6J and GAD67^*gfp*/+^ (Tamamaki et al. 2003)] were sedated with isoflurane (5%) and subsequently humanely killed by cervical dislocation for fresh foetal tissue collection or transcardially perfused with 4% paraformaldehyde (PFA) in 0.1 M PB (pH 7.4) for immunohistochemistry (*n* = 2–3 per experiment). The brains were dissected, post-fixed in 4% PFA overnight and subsequently cryoprotected in 30% sucrose for at least 48 h before being cryosectioned (Leica CM 1850 UV) at 50 µm thickness (free-floating sections) in phosphate-buffered saline (0.01 M PBS; pH 7.4) and processed for immunohistochemistry (Alpar et al. 2014). Neonatal mice [C57BL/6J and CCK^BAC/DsRed^::GAD67^*gfp*/+^ (Calvigioni et al. 2017)] were decapitated with their brains’ immersion fixed in 4% PFA overnight. Brains were cryoprotected in 30% sucrose and freeze–thaw cryosectioned at 20 µm thickness onto electrically charged glass slides (SuperFrost Plus).

### Primary neuronal and SH-SY5Y cultures

Foetal hippocampal neurons were obtained (Alpar et al. 2014) by harvesting embryos from C57BL/6NRj pregnant mice on embryonic day (E)18.5. Tissues were dissociated in 0.1% trypsin (Gibco) and DNAse Type 1 (Sigma) in DMEM (Invitrogen) for 5 min at 37 °C. Dissociated cells were washed with 0.4% BSA in DMEM followed by repeated washes in Neurobasal medium containing 100 U/mL penicillin and 100 µg/mL streptomycin (Gibco), 1 mM Glutamax (Gibco) and 1× B27 supplement (Gibco). Primary neurons were grown on poly-d-lysine (PDL; Sigma)-coated glass coverslips at 37 °C for 2, 4 and 7 days in vitro (DIV), and fixed with 4% PFA in PBS on ice for 15 min before being processed for immunohistochemistry.

SH-SY5Y human neuroblastoma cells were maintained in DMEM/F12 (1:1) containing 10% foetal bovine serum (FBS; Gibco), 1 mM sodium pyruvate (Gibco), 1 mM Glutamax and penicillin–streptomycin (as above). For differentiation, SH-SY5Y cells were seeded on PDL-coated glass coverslips in full growth medium for > 3 h. Subsequently, their differentiation was initiated with a growth medium containing 1% FBS and 10 µM all-trans retinoic acid (Sigma) for 7 DIV (Cheung et al. 2009). Differentiated cells were treated once (starting on day 7 and for 48 h) with synthetic β-amyloid (1–42), from 25 nM to 5 µM, custom-synthesized at the Department of Medical Chemistry, Szeged, Hungary (Bozso et al. 2010). Purified peptides were freshly dissolved in distilled water at room temperature, sonicated and stored at − 20 °C until use to prevent peptide aggregation (Bozso et al. 2010) at a stock concentration of 0.5 mM. β-Amyloid(1–42) was then freshly used at concentrations ranging from 25 nM to 5 µM (Lee et al. 2013). Cell density, to mark cell survival, was monitored on an EVOS XL Core microscope (Thermo Fisher), and quantified (20× magnification, 3–5 fields per condition) using Imaris ×64 (Bitplane, 8.3.0). Thereafter, SH-SY5Y cells were either fixed in 4% PFA in PBS and processed for immunocytochemistry or lysed for western blotting or qPCR.

### Gene expression analysis

mRNA extraction was performed from fresh frozen tissues and cultured cells using a SPLIT RNA extraction kit (Lexogen). One µg mRNA was converted to cDNA using a High Capacity cDNA Reverse Transcription kit (Thermo Fisher) on a T100 thermal cycler (Bio-Rad) and PCR amplified, as applicable, by mouse or human specific primers (Supplementary Table 2). PCR products were resolved on a 1.5% agarose gel and imaged on a ChemiDoc XRS^+^ system (Bio-Rad).

### Western blotting

Brain tissues and cultured cells were collected in lysis buffer containing (in mM): 25 HEPES (Sigma), 1 EDTA (Sigma), 6 MgCl_2_ (Sigma), 1 DTT (Sigma) and 1x protease inhibitor cocktail (EDTA Free; Roche) and disrupted by ultrasonication (5 pulses, 50% intensity; Bandelin Electronic). Protein concentrations were measured on a Nanodrop 2000 spectrophotometer (Thermo Fisher) and diluted, if necessary, to 1 µg/µL. Samples were resolved on a 13% SDS-containing gel cartridge (20 µg protein load) in an Amersham WB system (GE Healthcare). Primary antibody concentrations are referred to in Supplementary Table 3. Secondary anti-rabbit and anti-mouse antibodies were used at a concentration of 1:2500. Protein prevalence was analysed with the Amersham WB software package with total protein labelled by carbocyanine-5 in bulk as loading control. Fresh–frozen hippocampal tissue from adult subjects for antibody validation was provided by the Brain Bank of the Institute of Neurology, Medical University of Vienna, Austria, and processed similarly.

### Antibody generation, characterization and specificity

The GABA_A_ receptor subunit-specific antibodies used in this study were generated earlier in the laboratory of Dr. W. Sieghart (Hortnagl et al. 2013; Milenkovic et al. 2013; Sieghart 1995; Sieghart and Sperk 2002; Stojanovic et al. 2016). They were characterized extensively for specificity on knock-out brain lysates with western blot (Milenkovic et al. 2013), on knock-out brain sections with immunohistochemistry (Pirker et al. 2003; Zimprich et al. 1991) and displayed similar staining patterns as reported by others using alternative antibody sources (Waldvogel et al. 1999, 2004, 2008). Polyclonal antibodies against GABA_A_ receptor subunits α2, α3 and γ2 were raised in rabbits using maltose-binding protein (MBP) fusion proteins produced in *Escherichia coli* as previously described (Kasugai et al. 2010; Pirker et al. 2000; Sperk et al. 1997). Antisera were purified by affinity chromatography using the respective GST-subunit fusion proteins (Mossier et al. 1994). All three polyclonal antibodies were raised against sequences identical for rats and humans (C-terminal epitope of α2 and N-terminal epitope of α3) and showed strong homology between rat and human sequences (loop epitope of γ2). The commercially available monoclonal anti-α1 antibody (BD24; Millipore, 1:100) showed a similar cellular hippocampal staining pattern as previously reported (Waldvogel et al. 1999, 2004).

### Image analysis

For chromogenic immunostainings, images of entire glass slides were captured with a high-resolution digital slide scanner (NanoZoomer 2.0-HT: C9600-13, Hamamatsu Photonics). Single images were exported from digitalized slides using the compatible viewer software (NDP.view, NanoZoomer Digital Pathology Image) containing hippocampal structures: the dentate gyrus (DG), cornus ammonis 1–4 (CA1–4) subfields and subiculum. Individual layers within subfields (molecular layer (ML), granule cell layer (GL) and polymorph layer (PL) of DG; deep polymorph layer (dPL) of CA4; ventricular zone (VZ), intermediate zone (IZ), stratum oriens (SR), pyramidal cell layer (PyL), stratum radiatum (SR) and stratum lucidum (SL) of CA3; VZ, IZ, SO, PyL and SR for CA2 and CA1; VZ, IZ, PyL and ML for subiculum) were densitometrically analysed in detail. Cases of early development, where the CA1 subfield could not be separated from the subiculum, were used only to describe immunoreactivity distribution but were excluded from our statistical analysis. In addition, a white matter structure (internal capsule (IC)) was imaged to normalize signal density values. For each receptor subunit, three images at 40× magnification were exported from the CA1 and subiculum, while other smaller subregions in CA2, CA3 and the internal and external limbs of the DG were cropped out using a free-hand tool at 10× magnification. Quantitative analysis of immunoreactivity was performed in ImageJ (NIH, 1.50 g). After colour deconvolution to spectrally unmix the hematoxylin pigment (Colour Deconvolution Plugin), images were converted to 8 bit. The threshold defining immunopositivity was set uniformly for all images. Coverage of immunopositive structures was calculated for all exported images and expressed as percentage of the total surface area. Thus, potential technical bias due to different fixation and variable enzymatic DAB reactions were minimized. For immunofluorescence, images were captured with a Zeiss LSM880 laser-scanning microscope and linearly adjusted using the ZEN2010 software (Zeiss, Jena, Germany).

### Statistical analysis

Data were tested for outliers (boxplots), normal distribution (Lilliefors test) and homogeneity of variance (Levene’s test). ANOVA univariate general linear model (GLM) was applied to ranked values of each subunit (α1, α2, α3, and γ2) in all structures to determine differences between Down syndrome and control cases within individual age periods. Subsequently, post hoc analysis with Tukey’s range test (if equal variances and equal sample sizes were assumed) or Games–Howell test (if equal variances and equal sample size could not be assumed) was performed. A *p* value of < 0.05 was considered statistically significant. Values for α3 were not normalized, as this subunit is predominantly expressed in the white matter during foetal development (see Fig. 4), thus excluding the collection of tissue “background”. Statistical analyses were performed by using IBM SPSS version 21.0.

## Results

### General remarks

In this study, we investigated the distribution of developmentally prevalent γ2, α1, α2 and α3 GABA_A_ receptor subunits in the hippocampal CA regions (CA1–4), subiculum and dentate gyrus during development in human foetuses, infants and adults diagnosed with Down syndrome and age-matched controls (Supplementary Table 1). Since the migratory transition of neurons from the CA1 to the subiculum occurs gradually in period 1 (for a description of developmental periods see above), these two regions were designated as CA1/subiculum for that time (Arnold and Trojanowski 1996). To prevent stochastic staining variability (‘edge effects’), we divided the dentate gyrus into inner (DGi; continuation of CA3) and outer limbs (DGo; flanking the CA4).

### GABA_A_R γ2

**Fig. 1:**
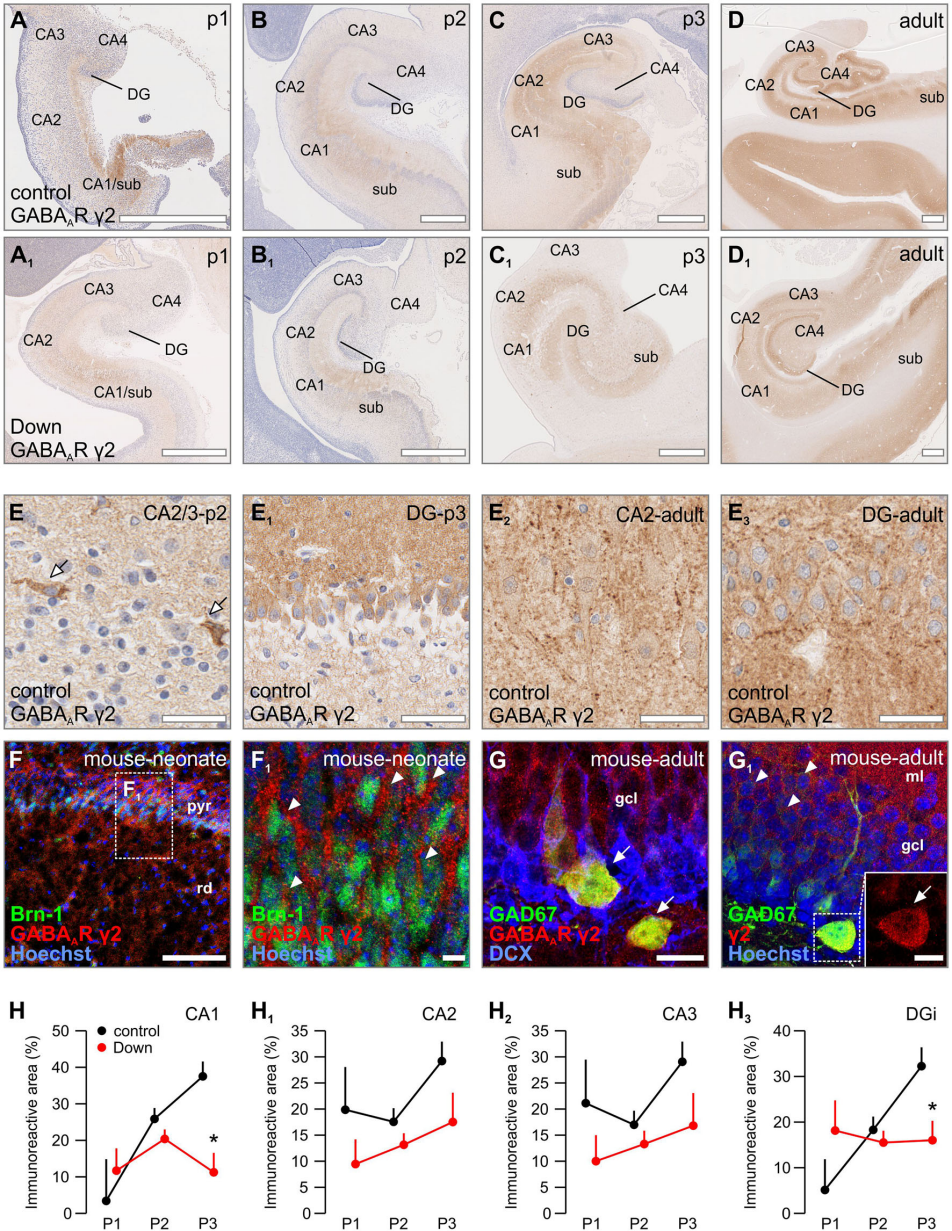
Distribution of GABA_A_R γ2 in normal vs. Down syndrome cases. **a**–**d**_1_ Overview images of foetal and adult hippocampi from representative Down syndrome cases and age-matched controls. **e**–**e**_3_ GABA_A_R γ2 immuno-labelling was found on pyramidal cells and interneurons (*arrows*, **e**–**e**_**1**_), with the most prominent staining of the neuropil in the dentate gyrus in adulthood (**e**_**2**_, **e**_**3**_). **f**, **f**_1_ Young pyramidal cells (arrowheads, Brn-1^+^) are positive for γ2 subunits in the neonatal mouse. **g**, **g**_**1**_ Interneurons (arrows, GAD67^+^) negative for double cortin (DCX) in the granular cell layer harbour γ2 subunits. **h**–**h**_**3**_ Analysis of immunoreactivity in the CA1, CA2, CA3 and inner dentate gyrus region, of GABA_A_R γ2 subunits throughout development in Down syndrome cases. *p < 0.05. Scalebars = 1 mm (**a**, **a**_**1**_, **b**, **b**_**1**_, **c**, **c**_**1**_) 500 µm (**d**, **d**_**1**_, **f**); 100 µm (**e**, **e**_**2**_); 50 µm (**e**); 30 µm (**e**_**3**_, **f**_**1**_, **g**); 15 µm (**g**_**1**_)

In periods 1 and 2, moderate γ2 immunoreactivity was noted throughout the human hippocampus, with the most notable signal localized to the CA1/subiculum (Fig. 1a, b). Although the immunoreactivity comprised mainly of neuropil labelling, we observed numerous cell populations that expressed γ2 subunits already early during development (period 2: *n* = 16 cases): cellular immunoreactivity was first detected in the CA1/subiculum [including pyramidal cells and interneurons (Fig. 1e)], later attained by other hippocampal regions. In period 3 (*n* = 5 cases), many pyramidal cells in the subiculum and CA regions, as well as granule cells in the dentate gyrus were positive for γ2 subunits (Fig. 1e_1_). The increase in γ2 immunoreactivity peaked in adulthood (Fig. 1d), with the most prominent increase noted in the CA1–3 regions (Fig. 1e_2_). γ2 Immunoreactivity was also seen across all layers of the adult dentate gyrus and comprised both neuropil and cytoplasmic labelling (Fig. 1e_3_).

To verify the cellular identify of the observed γ2 staining patterns, we tested γ2 expression in the developing mouse hippocampus by means of multiple fluorescence immunohistochemistry. In neonates (*n* = 2), γ2 immunoreactivity was mainly localized to the pyramidal layer in the CA1 and co-localized with *Brn*-*1*, a transcription factor transiently expressed in developing pyramidal cells (Alvarez-Bolado et al. 1995) (Fig. 1f, f_1_). In the adult dentate gyrus (*n* = 2), γ2 immunoreactivity was mainly seen as neuropil labelling in the molecular layer and perisomatically in the granule cell layer. Intermingled interneurons, genetically co-labelled for GAD67 (*Gad1*) by in-frame GFP knock-in (Tamamaki et al. 2003), exhibited somatic γ2 immunoreactivity (*n* = 2; Fig. 1g, g_1_).

To further examine γ2’s subcellular localization, we cultured primary mouse neurons to identify its spatiotemporal distribution pattern during the developmental period encompassing neurite outgrowth, growth cone motility and synaptogenesis. Up to 4DIV, γ2 immunoreactivity was found indiscriminately on the perikarya, neurites and growth cones of pyramidal-like cells (Supplementary Fig. 1A, A_1_). In more mature cultures (7DIV) with pre-formed neuronal networks, γ2-positive pearl–lace-like punctae were found on neuronal perikarya and processes, and were opposed by parallel-running processes, likely axons, positive for vesicle associated membrane protein 2 (VAMP2), which participates in the docking and fusion of synaptic vesicles (Washbourne et al. 1995), as well as the vesicular GABA transporter (VGAT) (McIntire et al. 1997) (Supplementary Fig. 1D–D_1_). Occasional VGAT^+^ interneurons were found expressing somatodendritic γ2 subunits (Supplementary Fig. 1D_2_) in a punctate fashion.

The γ2 expression pattern in early developmental periods of Down syndrome cases, periods 1 (*n* = 4) and 2 (*n* = 17), was comparable to that observed in age-matched controls (Fig. 1a, b vs. a_1_, b_1_). However, the amount of γ2 immunoreactivity was generally reduced in the hippocampus. A significant loss of γ2 immunoreactivity was found in the subiculum (*F*_(1,37)_ = 6.627, *p* = 0.014), the CA1 (*F*_(1,38)_ = 15.008, *p* = 0.000), and both the internal limb (*F*_(1,30)_ = 7.181, *p* = 0.012) and the external limb (*F*_(1,30)_ = 5.337, *p* = 0.028) of the dentate gyrus (Fig. 1h, h_3_; Supplementary Fig. 3E, E_1_) of Down syndrome cases (*n* = 6 control vs. *n* = 5 Down syndrome) around birth (period 3). In turn, γ2 immunoreactivity in the CA2 and CA3 subfields was comparable between diseased and heathy cases (Fig. 1f_1_, f_2_).

### GABA_A_R α1

**Fig. 2:**
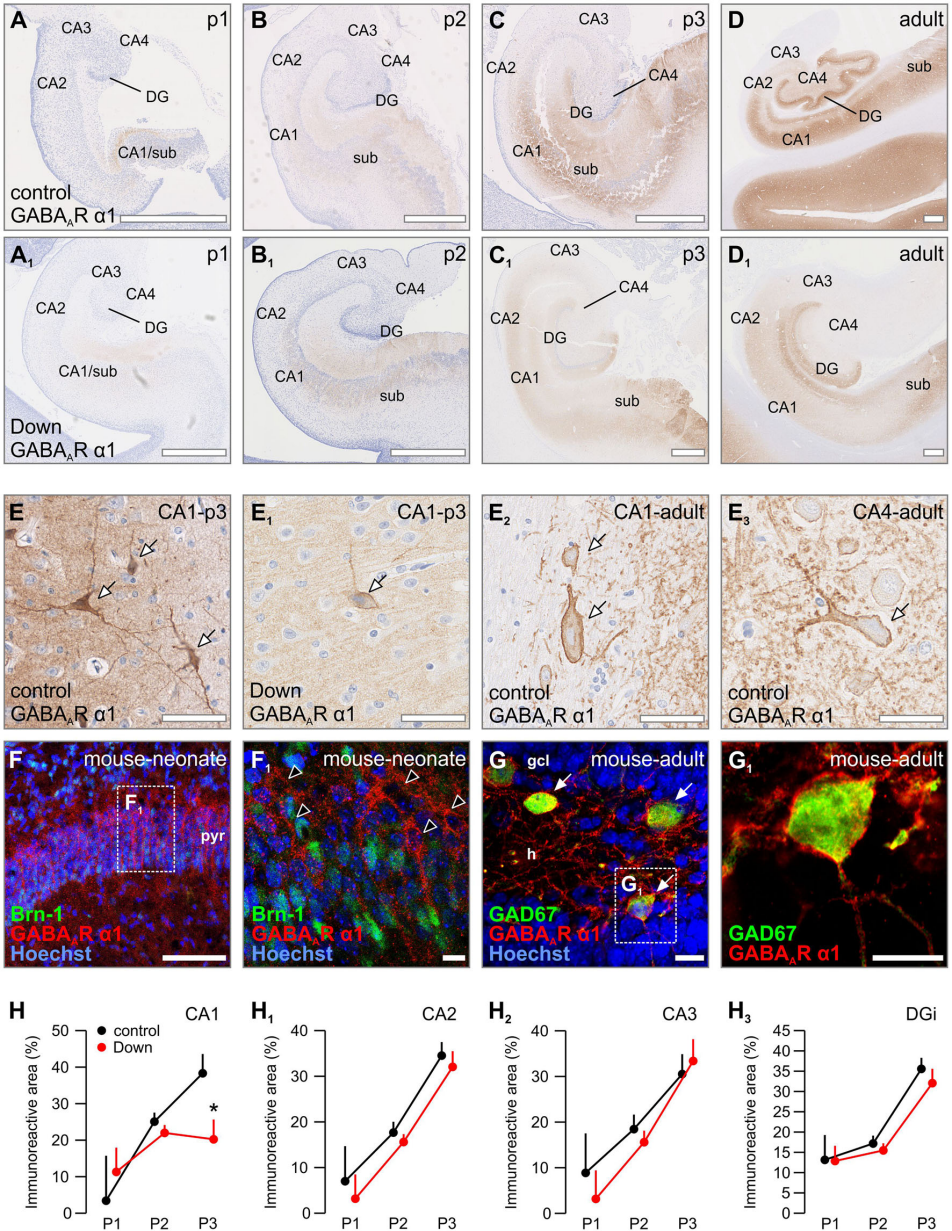
Distribution of GABA_A_R α1 in normal vs. Down syndrome cases. **a**–**d**_**1**_ Overview images of foetal and adult hippocampi from representative Down syndrome cases and age-matched controls. **e**–**e**_**3**_ Labelled pyramidal cells and interneurons (arrows) were found from period 3 onwards in hippocampal structures. Note the decrease in somatic and neuropil staining in Down syndrome (**e**_**1**_). **f**, **f**_**1**_ α1 subunits were found on Brn-1^−^ pyramidal neurons (open arrowheads). **g**, **g**_**1**_ Interneurons in the dentate gyrus express α1 subunits (arrows). **h**–**h**_**3**_ Quantifications revealed a significant loss of GABA_A_R α1 subunits in the CA1 region of period 3 only. *p < 0.05. Scalebars = 1 mm (**a**, **a**_**1**_, **b**, **b**_**1**_, **c**, **c**_**1**_) 500 µm (**d**, **d**_**1**_, **f**); 100 µm (**e**, **e**_**2**_, **e**_**3**_); 50 µm (**e**_**1**_); 30 µm (**f**_**1**_, **g**, **g**_**1**_)

In the hippocampi of human foetuses examined for periods 1 and 2, the α1 subunit was rarely detected (Fig. 2a, b). Instead, there was a prominent increase in α1 subunit immunoreactivity in period 3 (around birth) in most hippocampal subregions during normal development, except for CA3, with further enrichment until adulthood (Fig. 2c, d). In striking contrast to weak neuropil α1 immunoreactivity in periods 1 and 2, period 3 was characterized by α1 labelling in cell bodies and processes of the CA1–3 pyramidal layers, as well as the granule layer of the dentate gyrus (Supplementary Fig. 3A, A_1_). Towards adulthood, single cells reminiscent of pyramidal cells were decorated by α1 subunits in the pyramidal layer, predominantly in a patch-like manner along their plasmalemma. The other major group of cells positive for α1 had densely labelled cytoplasm and processes and was located in the outer part of the pyramidal layer just beneath the stratum oriens, resembling basket cells (*arrowheads*, Fig. 2d, e–e_2_). In the stratum radiatum, interneuron-like cells were found scattered among α1 immunoreactivity processes (Fig. 2e_3_). A similar pattern was observed in the subiculum (*data not shown*). The dentate gyrus exhibited strong neuropil immunoreactivity in its molecular layer and to a lesser extent in its granule cell layer in period 3 and particularly in adulthood (Supplementary Fig. 1B_2_, 1B_3_, 4A). Similar to other hippocampal regions and ages, strong immunoreactivity was mostly noted in interneuron-like cells (Supplementary Fig. 1B_2_, 1B_3_, 4A_1_).

Immunohistochemistry for the α1 subunit in neonatal mouse hippocampus showed that pyramidal cells positive for α1 in the pyramidal layer were mostly negative for *Brn*-*1* (Fig. 2f, f_1_). These cells layered above *Brn*-*1* ^+^ cells and are either maturing (down-regulated *Brn*-*1*) or a subpopulation positive for other transcription factors, such as *Brn*-*2* (Alvarez-Bolado et al. 1995). Interneurons defined by the expression of GAD67 in the CA1 and dentate gyrus of the mouse hippocampus were positive for the α1 subunit, being comparable to humans (Fig. 1g, g_1_).

The cellular distribution of α1 subunits was determined in mouse primary neuronal cultures. Labelling for α1 was not detected in DIV4 pyramidal-like neurons except minimal immunoreactivity on the proximal segment of their neurites (Supplementary Fig. 1B, B_1_). α1 labelling was not seen in growth cones either (Supplementary Fig. 1B_1_). At 7DIV, densely packed post-synaptic α1 immunoreactivity puncta apposed VAMP2 and VGAT immunoreactivities along somas and dendrite-like processes of pyramidal-shaped neurons (Supplementary Fig. 1e, e_1_). Interneurons (with VGAT^+^ axons) were occasionally found decorated by α1 puncta along their somatodendritic axis (Supplementary Fig. 1e_2_). This pattern is similar to that of the γ2 subunit, reminiscent of extrasynaptic receptors involved in tonic GABA signalling (Milenkovic et al. 2013).

Although the layer-specific and region-specific distribution of α1 subunits in the hippocampus of subjects with Down syndrome was comparable to those of normal foetuses, we noted an overall reduction in α1 immunoreactivity (Fig. 2a–d vs. a_1_–d_1_). This reduction was mainly due to the loss of neuropil immunoreactivity, while neuronal perikarya retained labelling with an intensity equalling control cases (Fig. 2e_1_). Interestingly, morphologically identified interneurons seemed to be the least affected by this reduction in period 3 (Fig. 2e_1_) with statistically significant decreases observed only in the neuropil of the CA1 subfield (*F*_(1,40)_ = 5.494, *p* = 0.024) (Fig. 2f). Indeed, the majority of Down syndrome cases in period 3 showed a significant decrease in α1 immunoreactivity in the CA1 (Fig. 2c_1_) which persisted until adulthood (Fig. 2d_1_). In other CA and DG regions, α1 immunoreactivity was in general weaker (Supplementary Fig. 3F, F_1_), but not statistically significant in any of the investigated periods (Fig. 2h–h_3_).

### GABA_A_R α2

**Fig. 3:**
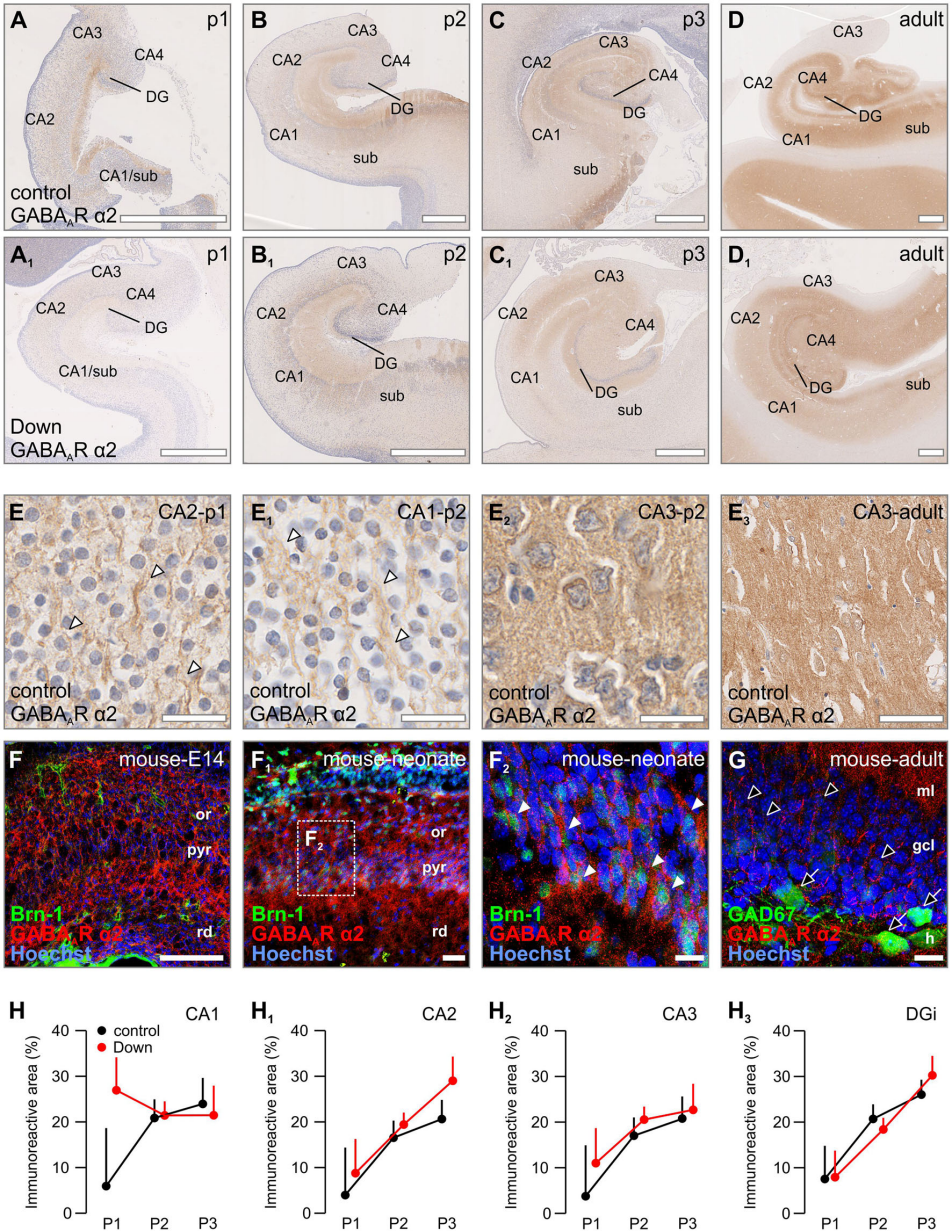
Distribution of GABA_A_R α2 in normal vs. Down syndrome cases. **a**–**d**_**1**_ Overview images of foetal and adult hippocampi from representative Down syndrome cases and age-matched controls. **e**–**e**_**3**_ GABA_A_R α2 immunoreactivity was found throughout all ages, labelling wavy processes (arrowheads, **e**, **e**_**1**_) and neuropil (**e**_**2**_, **e**_**3**_). **f**–**f**_**2**_ α2 Subunits were found throughout the embryonic (E14) and neonatal hippocampus on Brn-1^+^ pyramidal neurons (arrowheads, **f**_**2**_). **g** Interneurons and granule cells in the dentate gyrus were negative for α1 subunits (open arrows and arrowheads, respectively). **h**–**h**_**3**_ A trend towards an increase of GABA_A_R α2 immunoreactivity was only found in the CA1 region during period 1. Scalebars = 1 mm (**a**, **a**_**1**_, **b**, **b**_**1**_, **c**, **c**_**1**_) 500 µm (**d**, **d**_**1**_); 200 µm (**f**); 50 µm (**e**_**3**_, **f**_**1**_); 30 µm (**e**, **e**_**1**_, **e**_**2**_, **g**); 15 µm (**f**_**2**_)

In period 1, the α2 subunit was predominantly observed on a fine network of fibres throughout all subregions studied in the human hippocampus (Fig. 3a). For instance, α2 immunoreactivity was enriched in wavy processes passing along cells from period 1 onward in the early pyramidal layer (Fig. 3a–d, e, e_1_). During period 2, an increasing number of α2-expressing cells with neuron-like morphology was noted in the dentate gyrus, CA layers and the subiculum, and exhibited cytoplasmic α2 immunoreactivity (Fig. 3b). In the developing mouse, this cytoplasmic immunoreactivity was seen at E14 throughout the entire primordial hippocampus (Fig. 3f). Processes coursing between cells, comparable to the human foetus (Fig. 3e, e_1_), appeared in the neonatal mouse emanating from *Brn*-*1*-positive cells, suggesting immature pyramidal neurons (Fig. 1f_2_).

During successive stages of human development, immunoreactivity on processes became gradually reduced (Fig. 3e_1_), leaving a strong neuropil labelling, as well as numerous cells (including pyramidal-like and granule-like cells), in the adult CA regions and dentate gyrus (Fig. 3e_2_, e_3_). Notably, α2 immunoreactivity seemed to be more pronounced in the inner 1/3 of the molecular layer of the adult DG (Supplementary Fig. 3B, B_1_). In the adult mouse, α2 immunoreactivity was similarly decreased in the pyramidal layer, leaving a perisomatic staining pattern on pyramidal cells, but was not found expressed by GFP-containing interneurons on a GAD67^gfp/+^ background (Fig. 3g).

Similar to the patterns found in vivo, α2 subunits were seen on perikarya, processes and growth cones of cultured mouse pyramidal-like neurons at 4DIV (Supplementary Fig. 1C, C_1_). By 7DIV in maturing cultures, α2 immunoreactivity was mainly restricted to postsynapse-like structures on pyramidal-like neurons apposing VGAT and VAMP2 boutons (Supplementary Fig. 2E–E_2_) with minimal IMMUNOREACTIVITY remaining on the somatodendritic compartment relative to earlier time points.

The overall distribution of α2 subunits in Down syndrome was similar compared to the control cases (Fig. 3a–d vs. a_1_–d_1_). The majority of hippocampal subregions (the dentate gyrus, CA2, CA3, CA4) presented neither significant differences in immunoreactivity nor cellular distribution (Fig. 3h–h_3_). The initial higher neuropil immunoreactivity in Down cases reached levels comparable to those in control cases in period 2 (Fig. 3b, b_1_, f). For all other areas (Supplementary Fig. 3G, G_1_), the immunoreactivity measured was generally weaker in Down syndrome albeit not reaching statistical significance.

### GABA_A_R α3

**Fig. 4:**
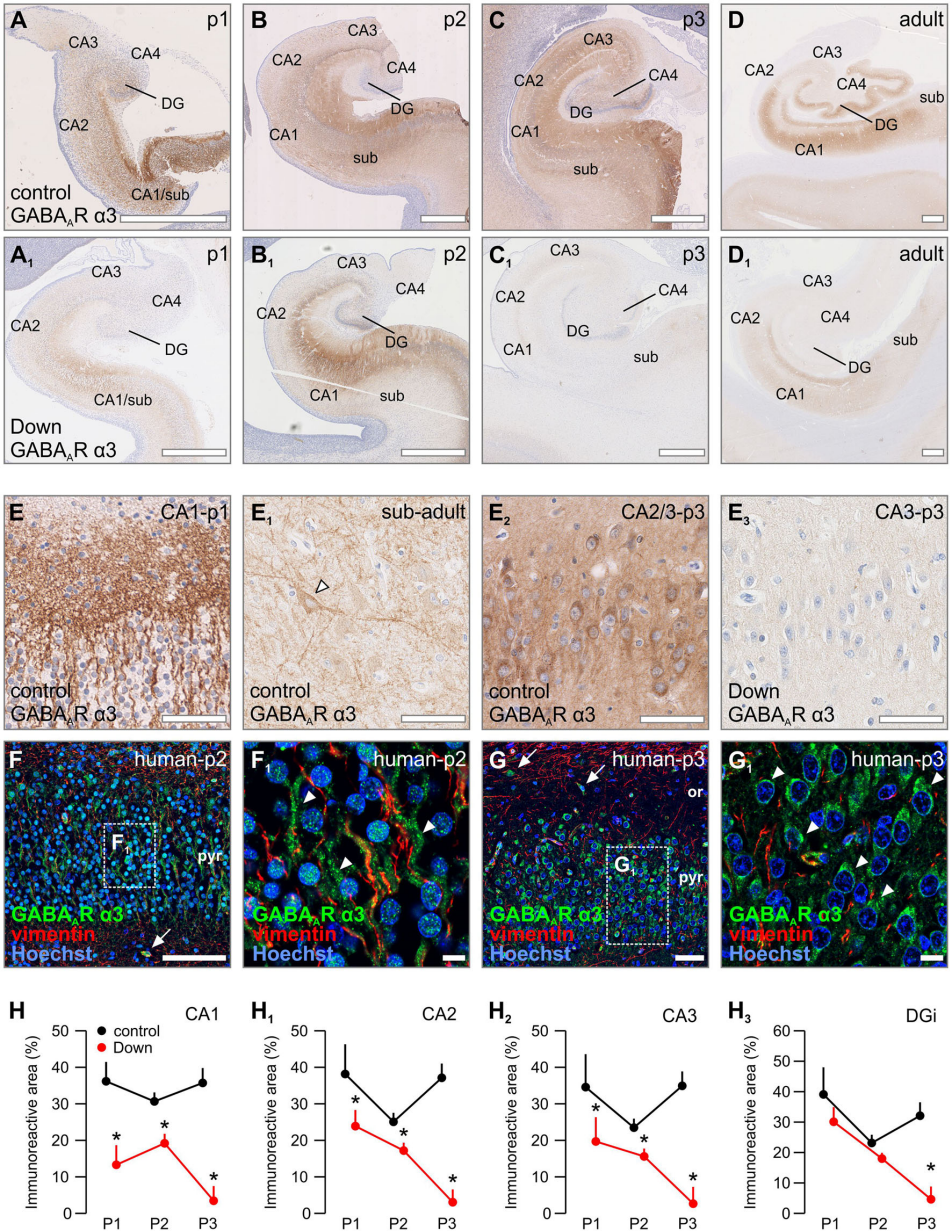
Distribution of GABA_A_R α3 in normal vs. Down syndrome cases. **a**–**f**_**1**_ Overview images of foetal and adult hippocampi from representative Down syndrome cases and age-matched controls. **e**–**e**_**3**_ Labelling for GABA_A_R α3 was found as early as period 1 in immature pyramidal-like neurons in the pyramidal layer of the CA1 (**e**). Immunoreactivity was gradually lost into adulthood, with interneurons remaining positively labelled (arrowhead, **e**_**1**_). The overall neuropil and pyramidal layer staining of the hippocampus in Down syndrome was generally lower (**e**_**2**_ vs. **e**_**3**_). **f**, **f**_**1**_ α3 Subunits were found in the pyramidal layer of the human CA1 (period 2) coursing along vimentin^+^ radial glia processes. **g**, **g**_**1**_ α3 Subunit immunoreactivity was reduced to a somatic pattern in later periods (period 3). **h**–**h**_**3**_ Quantifications reveal a significant loss of GABA_A_R α3 subunits in Down syndrome over all ages and all structures measured. *p < 0.05. Scalebars = 1 mm (**a**, **a**_**1**_, **b**, **b**_**1**_, **c**, **c**_**1**_) 500 µm (**d**, **d**_**1**_, **f**); 100 µm (**e**, **e**_**1**_, **g**); 50 µm (**e**_**2**_, **e**_**3**_); 30 µm (**e**_**3**_); 15 µm (**f**_**1**_, **g**_**1**_)

In contrast to the distribution of α1 and α2 subunits, the α3 subunit was found at higher levels in the developing human hippocampus and parahippocampal gyrus (Fig. 4a–d) than in adults. From period 1 on, the α3 subunit was indiscriminately expressed in all subfields. These included: (1) processes in the pyramidal layer, as well as the neuropil, of the subiculum and CA regions (Fig. 4e; Supplementary Fig. 3D–D_3_), (2) pyramidal-like and interneuron-like somata in the subiculum and CA regions (Fig. 4e_1_, e_2_) and (3) granule cells in the dentate gyrus (Supplementary Fig. 3C, C_1_). Neurons expressing α3 subunits showed somatic immunoreactivity, which continued in wavy neurites that were associated with vimentin-positive processes, a marker for radial glia (Dahl et al. 1981), suggestive of prospective axons during pathfinding (Fig. 4f, f_1_). Bulk immunoreactivity progressively decreased in the subiculum, CA2, CA3 and CA4 with advancing gestational age (Fig. 4c, d). This was primarily due to reduced α3 immunoreactivity in processes, whereas strong and often punctuate somatic α3 immunoreactivity was retained in pyramidal cells in period 3 (Fig. 4g, g_1_). Distinct from other developmental ages, the dentate gyrus exhibited one of the strongest α3 immunoreactivity in adults, with immunolabeling in the inner 1/3 of its molecular layer, corresponding to input fields of the local inhibitory circuitry. Furthermore, the majority of granule cells co-expressed α3 subunits, as well (Supplementary Fig. 3D, D_1_). These data suggest a role for α3 subunits in developmentally regulated GABA signalling in emergent neuronal networks during human foetal development.

Throughout development in Down syndrome, we noted substantially lower levels of both α3 immunoreactivity intensity and its more restricted layer distribution (Fig. 4a–d vs. a_1_–d_1_) relative to controls. In period 1, α3 immunoreactivity was significantly lower in the CA1 of Down syndrome cases (*F*_(1,39)_ = 4.582, *p* = 0.039) (Fig. 4h). In period 2, levels of immunoreactivity were still significantly lower than in control cases in the subiculum (*F*_(1,39)_ = 19.118, *p* < 0.001), CA1 (*F*_(1,39)_ = 12.121, *p* = 0.001), CA2 (*F*_(1,34)_ = 6.478, *p* = 0.016) and CA3 (*F*_(1,32)_ = 4.985, *p* = 0.03) (Fig. 4h–h_3_; Supplementary Fig. 3H). Furthermore, the adjacent intermediate and marginal zones in the CA regions exhibited a reduction in expression patterns, too. In period 3, we observed a significant and subtotal loss of α3 immunoreactivity, leaving barely any immunoreactivity detectable throughout the hippocampus in Down syndrome cases (Fig. 4c_1_); the subiculum (*F*_(1,39)_ = 19.919, *p* < 0.001), CA1 (*F*_(1,39)_ = 31.205, *p* < 0.001), CA2 (*F*_(1,34)_ = 43.841, *p* < 0.001), CA3 (*F*_(1,32)_ = 24. 975, *p* < 0.001), the internal limb of the dentate gyrus (*F*_(1,34)_ = 20.741, *p* < 0.001) and external limb of the dentate gyrus (*F*_(1,34)_ = 18.879, *p* < 0.001) (Supplementary Fig. 4D–D_1_, H–H_3_). Noteworthy, α3 immunoreactivity on interneuron-like cells seemed more robust than pyramidal cells, which were devoid of α3 immunoreactivity around periods 1 and 2 (Fig. 4b, b_1_, e_2_, e_3_). In contrast, α3 expression in interneurons was reduced at much later developmental stages (Fig. 4e_1_), while lower α3 levels on interneurons were observed in adult Down syndrome cases (Fig. 4d, d_1_).

### α3 Subunits in neurochemically defined hippocampal neurons

**Fig. 5:**
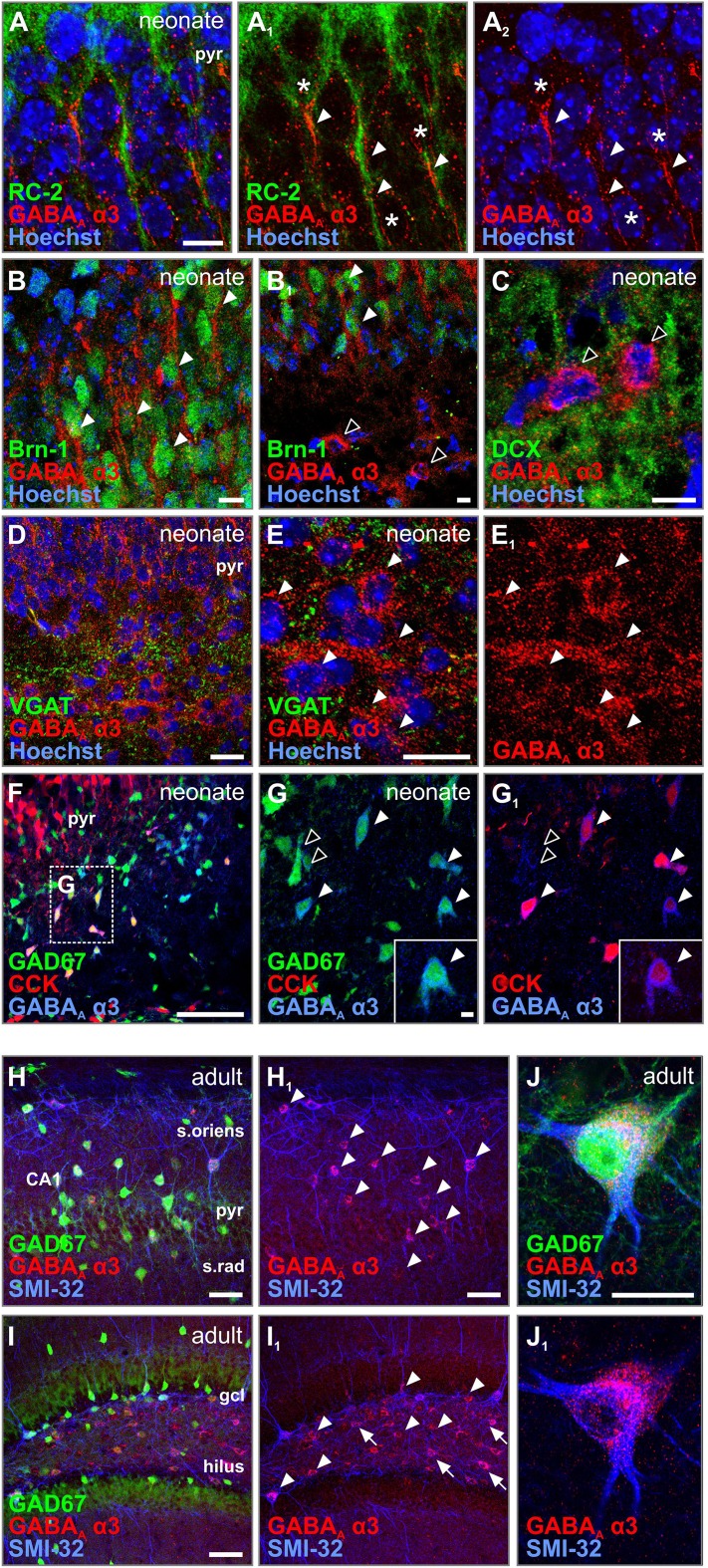
GABA_A_R α3 localizes to pyramidal cells and interneurons in the foetal mouse brain. **a**–**a**_**2**_ In the pyramidal layer, GABA_A_R α3 subunits were found on somata (asterisks) and processes (arrowheads) adjacent to radial glia (RC-2^+^), indicative of young pyramidal cells. **b**, **b**_**1**_ α3 Subunits were expressed on Brn-1 containing pyramidal cells (arrowhead), but not on interneuron-like cells (open arrowheads). **c** Interneuron-like cells were negative for the migration marker DCX (open arrowheads). **d**–**e**_**1**_ In the CA1, GABA_A_R α3 immunoreactivity was found on migrating interneuron-like cells embedded in a VGAT^+^ meshwork (arrowheads, **e**–**e**_**1**_). **f**–**g**_**1**_ The dual-transgenic mouse GAD67-GFP::CCK-DsRed revealed that these cells are indeed interneurons (GAD67^+^; open arrowheads), with a subpopulation of CCK-positive cells (solid arrowheads). **h**–**j**_**1**_ In the adult, GABA_A_R α3 staining was limited to somata of interneurons (arrowheads) in the CA1 region (**h**, **h**_**1**_) and the dentate gyrus (**i**–**j**_**1**_). Occasional cells were GAD67-negative, most likely CCK-containing interneurons (arrows). Scalebars = 500 µm (**f**); 100 µm (**d**, **h**, **i**); 50 µm (**e**, **j**); 30 µm (**a**–**c**); 15 µm (**g**)

Since the α3 subunit’s expression and distribution were most affected in Down’s syndrome and this subunit is the least described during brain development, we next pursued its (sub)-cellular localization by multi-colour fluorescence immunohistochemistry and high-resolution confocal laser-scanning microscopy in foetal and adult *mouse* tissues. Similar to its human developmental pattern (Fig. 1g–h_2_), α3 subunits were mostly found in the neonatal (comparable to period 3) pyramidal layer (Fig. 5a). Here, α3 subunits were found on the somata and proximal processes in close proximity to radial glia fibres (RC2^+^) of neurons that co-labelled for *Brn*-*1* (Fig. 5b, b_1_), indicating that young pyramidal cells can harbour α3 expression during their developmental trajectory.

In addition, α3 subunits were found on bipolar cells [negative for the neuronal migration marker doublecortin (DCX) (Gleeson et al. 1998)], surrounded by a meshwork of VGAT^+^ puncta (Fig. 5c–e_1_). To validate if these bipolar cells are either GAD67^+^ and/or cholecystokinin (CCK)-containing interneurons (Klausberger and Somogyi 2008), we utilized a novel CCK^BAC/DsRed^::GAD67^*gfp*/+^ dual-colour reporter mouse (Calvigioni et al. 2017). Almost all α3^+^ cell bodies co-localized with GAD67, with a subpopulation being also positive for CCK (Fig. 5f–g_1_), reinforcing that morphologically differentiating interneurons can express α3. Comparable to its human developmental distribution (Fig. 3d), α3 immunoreactivity was reduced in the adult mouse hippocampus: residual punctate labelling in the pyramidal layer and mainly GFP^+^ interneurons were noted (Fig. 5h–j_1_). These data suggest that interneurons retain their α3 expression, whereas pyramidal cells down-regulate this GABA_A_ subunit during hippocampal development.

**Fig. 6:**
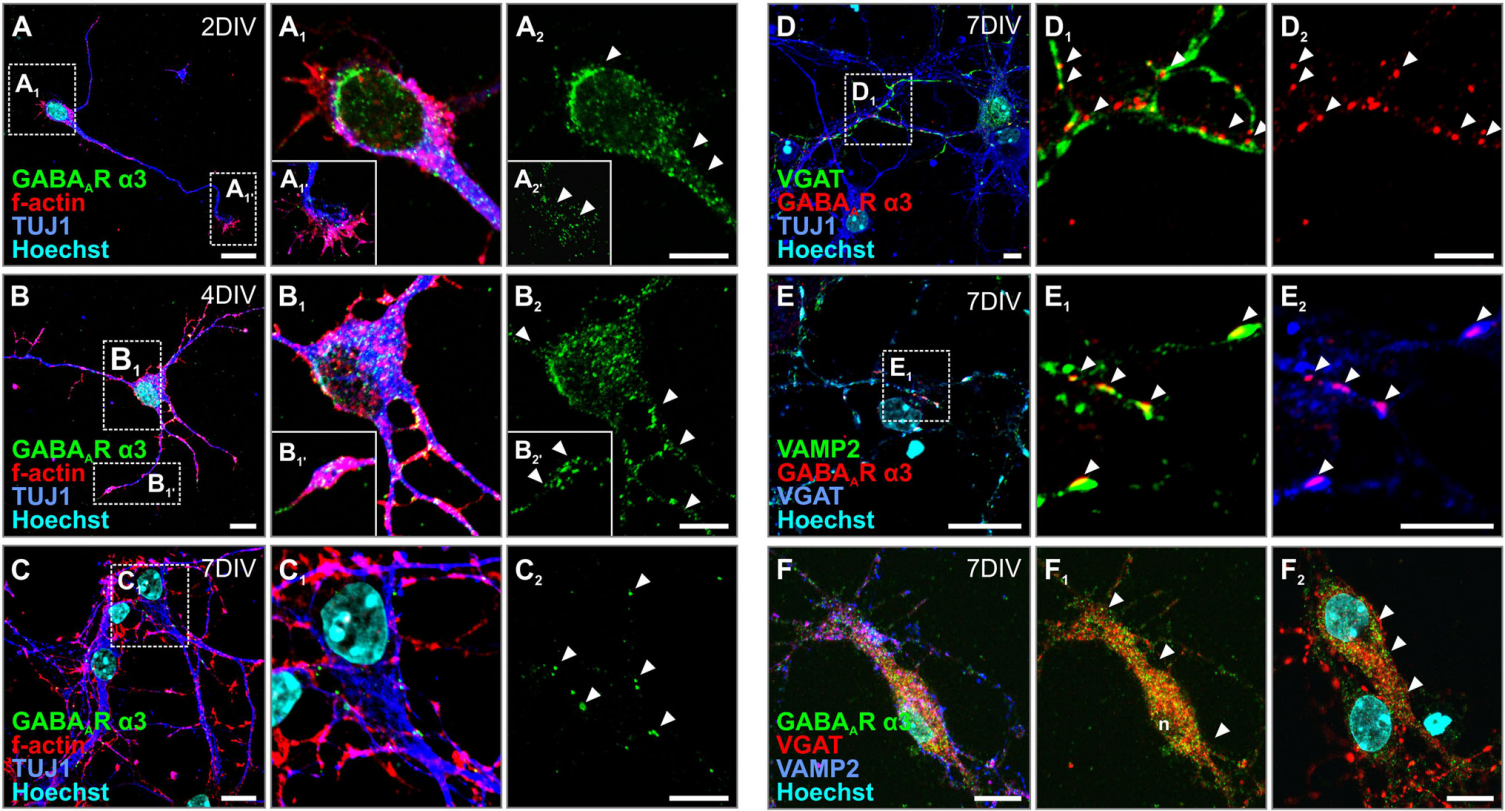
GABA_A_R α3 redistributes during mouse neuronal differentiation. **a**–**b**_**2**_ In cultured mouse primary hippocampal neurons, GABA_A_R α3 subunits are localized to somata, neurites and growth cones (f-actin-positive), up to 4 days in culture (arrowheads). **c**–**c**_**2**_ When neuronal networks start to form, GABA_A_R α3 subunits redistribute with a punctate pattern on neurites and somatas (arrowhead*s*). **d**–**e**_**2**_ GABA_A_R α3 subunits are found adjacent to VGAT^+^ puncta (**d**_**1**_, **d**_**2**_) and the presynaptic vesicular marker VAMP2 (**e**_**1**_, **e**_**2**_), indicating that GABA_A_R α3 relocalizes to neurotransmitter release sites upon network formation. **f**–**f**_**2**_ Intermittent interneurons, VGAT^+^, were found decorated with GABA_A_R α3 subunits (arrowheads). Scalebars = 50 µm (**e**, **j**); 30 µm (**a**, **a**_**2**_, **b**, **b**_**2**_, **c**, **c**_**2**_, **e**, **f**, **f**_**2**_); 15 µm (**d**, **d**_**2**_, **e**_**2**_)

We then further dissected α3 subcellular distribution in cultured mouse primary neurons with an emphasis on its redistribution and/or down-regulation during neuronal maturation. At 2DIV, α3 immunoreactivity was resolved along the soma, processes and growth cones of pyramidal-like cells (Fig. 6a–a_2_). After 4DIV, α3 immunoreactivity was still retained in actin-rich growth cones, fostering a concept on α3 subunit contributions to GABA-mediated axon guidance (Fig. 6b–b_2_) (Ageta-Ishihara et al. 2009). At 7DIV, when neuronal networks first appeared, α3 immunoreactivity was only sparsely detected on somas and processes (Fig. 6c–c_2_). Yet, α3 subunits concentrated in apposition to VGAT^+^ presynapses, which also contained VAMP2 (Fig. 6d–e_2_), thus marking bona fide synapses. Moreover, α3 IMMUNOREACTIVITY was occasionally seen in cells adopting glia-like morphology (Supplementary Fig. 2A–A_2_). These results imply that α3 subunits in developing neurons are either progressively down-regulated or recruited to post-synaptic sites. Interneurons are recognized as the sole cellular component of the cortical circuitry to retain α3 expression (Fig. 5f–h_1_) perisomatically until adulthood.

### Cellular exposure to β-amyloid leads to down-regulation of α3 subunits

To seek a more mechanistic view on the down-regulation of α3 subunit expression in Down syndrome, we selected amyloid precursor protein 1 (APP) as a candidate for upstream regulation due to its presence on chromosome 21 and for it being implicated in driving neuronal differentiation and synaptogenesis (Korenberg et al. 1989; Wang et al. 2009). In addition, inhibition of γ-secretase, a multiprotein complex with enzymatic activity to cleave APP and generate full-length β-amyloid (1–42), restores neurogenesis and synaptogenesis in a mouse model of Down syndrome (Giacomini et al. 2015).

**Fig. 7:**
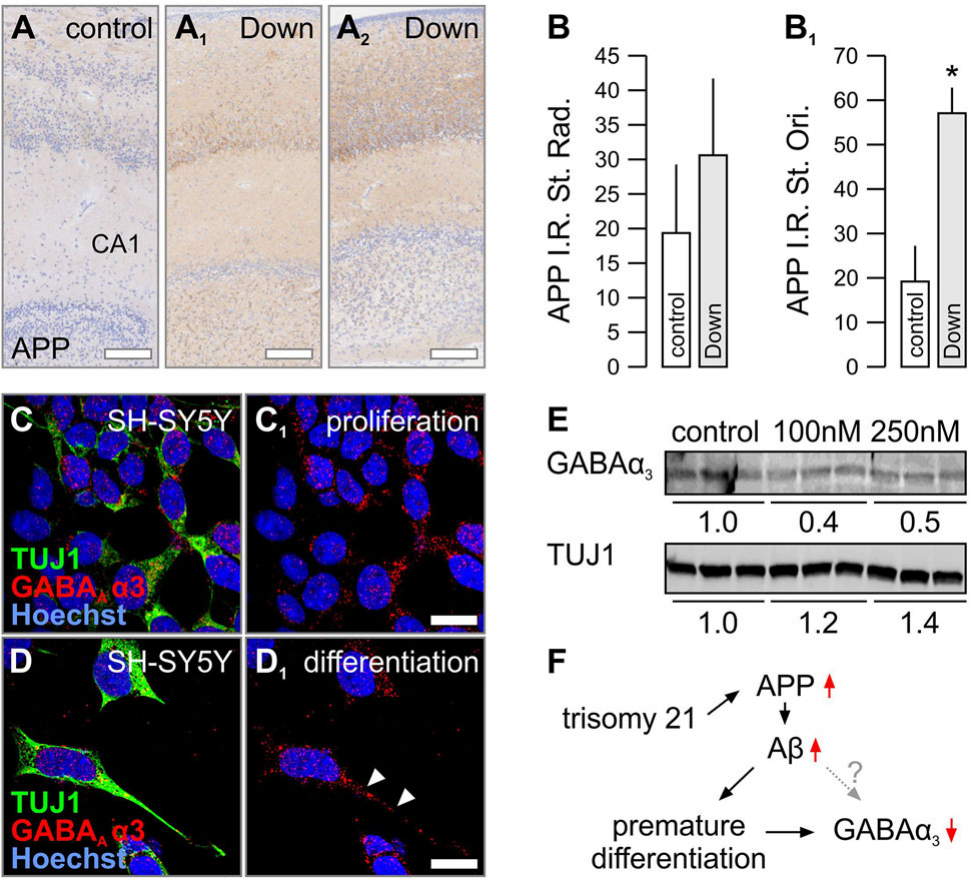
Beta amyloid is implicated in the premature differentiation of human neuronal-like cells. **a**–**b**_**2**_ APP levels in the hippocampus are significantly increased in Down syndrome patients (period 1/2, *n* = 5–6). **c**, **c**_**1**_ In undifferentiated human SH-SY5Y cells, α3 subunits are localized to the cytoplasma (arrowheads). **d**, **d**_**1**_ Upon differentiation, TUJ1 levels increase and α3 subunits appear on elongating TUJ1^+^ processes (arrowheads). **e** Non-toxic concentrations of beta amyloid(1–42) down-regulate α3 subunits while increasing TUJ1 levels. **f** Proposed mechanism of premature maturation of brain circuitry. *p < 0.05. Scalebars = 200 µm (**a**, **a**_**1**_, **a**_**2**_); 50 µm (**c**_**1**_, **d**_**1**_)

We found that APP was significantly up-regulated in the stratum oriens of the pyramidal layer of the CA1 region during periods 1/2 (*p* < 0.01; *n* = 5 control vs. *n* = 5 Down cases), with a coincidently marked increase in the stratum radiatum in cases with Down syndrome (Fig. 7a–b_2_). Since plasma β-amyloid (1–42) levels are increased in young adults with Down syndrome and associate with accelerated ageing in these patients (Obeid et al. 2016), we hypothesized that an increase of soluble β-amyloid, produced by γ-secretase-mediated proteolytic cleavage of excess APP, might disrupt the morphogenesis of developing neurons (Freude et al. 2011) and precipitate regulatory changes to dictate α3 subunit availability. Since cultured primary neurons express low amounts of α3 subunits, especially when neuronal networks are formed (Fig. 6b, c), we relied on human SH-SY5Y neuroblastoma cells as a cellular model to test our hypothesis. SH-SY5Y cells express α3 subunits at high mRNA and protein levels (Supplementary Fig. 4A, A_1_) and undergo morphological changes when exposed to β-amyloid (J. Mulder and T. Harkany, *unpublished data*).

Differentiation of SH-SY5Y cells was initiated by application of retinoic acid (Cheung et al. 2009) and verified by their up-regulation of β-III-tubulin (TUJ1), a cytoskeletal marker of immature neurons (Fig. 7c–d_1_; *n* = 3 coverslips). Coincidently, α3 subunits appeared in processes of SH-SY5Y cells undergoing retinoic acid-induced differentiation (Fig. 7d, d_1_). After application of fresh β-amyloid(1–42) at concentrations (100–250 nM) that did not affect the survival of cells undergoing retinoic acid-induced differentiation in our culture paradigm (Supplementary Fig. 4B–C_2_), TUJ1 levels significantly increased relative to untreated yet differentiated controls (Fig. 7e; *n* = 3 each). This response was anti-parallel with α3 subunit levels (that decreased; Fig. 7e). When applying μM concentrations of β-amyloid(1–42), we noted cytotoxicity (Harkany et al. 2000a, b) (Supplementary Fig. 4B–C_2_), indicating that there is a narrow developmental window in which β-amyloid(1–42) might exert morphogenic, perhaps even differentiation-promoting effects on developing neurons, if a tandem of TUJ1 and α3 are seen as read-outs. In sum, our data suggests that β-amyloid, a pathogenic outcome of increased APP levels, in Down syndrome could contribute to the remodelling of GABA synapses by altering their subunit composition (Fig. 7f).

## Discussion

In this study, we present the first comprehensive description of the expression pattern of α1, α2, α3 and γ2 GABA_A_ receptor subunits in the human hippocampus of Down syndrome cases and age-matched controls along a broad developmental trajectory. Although the existence of functional GABA_A_ receptors in primate and human brains was demonstrated by visualization of benzodiazepine binding sites (Reichelt et al. 1991; Shaw et al. 1991), there is a surprising gap of knowledge at the protein level due to the lack of appropriate tools. Therefore, we applied novel knock-out-tested antibodies against GABA_A_R subunits to address this issue.

We find differential maturation of α1 and α2 subunits in the hippocampus. Similar to data from animal models, α1 seems to have late expressional onset in the human brain (Fritschy et al. 1994). In contrast, an abundance of α3 subunits throughout the developmental hippocampus was observed with significantly diminished levels in the adult. GABA_A_Rs have been implicated in the migration of post-mitotic neurons, including both pyramidal cells (Behar et al. 2000) and interneurons (Cuzon et al. 2006), postulating a central role for this subunit to control migration and differentiation. Our finding that α3 subunits are robustly expressed during development on both interneurons and pyramidal cells in vivo, as well as on actin-rich motile growth domains in vitro, reinforces a non-synaptic trophic and/or guidance role for GABA signalling through α3 subunits in the developing hippocampus. In addition, the α3-subunit seems to be exchanged perinatally to other subunits (i.e. α1 and α2) in the human foetal brain, which might account for the change in electrophysiological properties that GABA_A_R signalling undergoes during normal brain development (Owens et al. 1999). While, the γ2 subunit showed widespread expression in the hippocampus, we observed a striking similarity in expression pattern between α3 and γ2 subunits in the CA1 and subiculum, suggesting their probable functional co-occurrence. The temporal co-expression of these two subunits suggests that these possible complexes are destined for synaptic terminals, since γ2 is classified as the post-synaptic subunit and is involved in the trafficking of the complex towards the synapse (Vithlani et al. 2011). Noteworthy, we did not detect significant amounts of α3 in the DG, suggesting different assembly partners for γ2 in this region.

In the hippocampus of foetal Down syndrome subjects, our data indicates complex expression profiles coincidently affecting α1, α3 and γ2 subunits. For instance, α1 and γ2 subunits showed selective depletion in the CA1 and subiculum, whereas in other hippocampal areas these changes were not as obvious. Interestingly, the distribution of α2 subunits in the hippocampus bore a striking resemblance to the γ2 subunit, both of them exhibiting a subtotal depletion in the CA1 and subiculum in period 3. In contrast, the α3 subunit demonstrated the most robust changes in Down syndrome cases. A strong reduction was observed throughout all investigated time periods with an overall lower expression and an almost complete loss from period 3 onwards. Most adult animal studies focused on pharmacological manipulation of α5 subunit containing GABA_A_Rs (Braudeau et al. 2011; Fernandez et al. 2007; Martinez-Cue et al. 2013), due to their involvement in cognition (Redrobe et al. 2012; Rudolph and Mohler 2014; Wang et al. 2012). We were unable to reliably probe the α5 subunit with the antibodies available to us in developmental tissues, revealing only a weak punctate staining that we deemed unspecific due to a lack of regional and temporal patterns (*data not shown*). Therefore, we only focussed on GABA_A_R subunits whose consistent staining pattern we deemed specific.

Given the fact that GABA_A_Rs are thought to provide a stop signal for migrating cells in the cortex (Behar et al. 2000), a disturbance in GABA_A_R subunits would suggest alterations to neuronal migration in Down syndrome. Indeed, a change in neurogenesis and reduced neuronal number in the cortex have been described in Down syndrome before (Becker et al. 1991; Golden and Hyman 1994; Ross et al. 1984; Wisniewski et al. 1984). Since we localized α3 subunits to motile growth domains on both pyramidal cells and interneurons, our data suggest that a premature down-regulation of α3 subunits might contribute to those observed changes in neuronal migration, especially if this reduction leads to altered GABA_A_R-mediated currents or second messenger signalling systems (Luscher et al. 2011). Thus, a premature redistribution of subunits could eventually lead to improper neuronal placement and differentiation, and disturb prenatal giant depolarizing potentials which are important for the maturation of synapses (Ben-Ari et al. 2007) overall, resulting in cognitive deficits.

A concurrent comorbidity in Down syndrome is the early development of β-amyloid plaques in the adult brain and Alzheimer’s-like neurological abnormalities (Masters et al. 1985; Motte and Williams 1989). Due to the position of APP on chromosome 21, APP is thought to elevate levels of full-length β-amyloid, leading to early plaque deposits. In addition, inhibitors of γ-secretase, restored neurogenesis and synaptogenesis in a mouse model of Down syndrome (Giacomini et al. 2015). However, β/γ secretase cleavage of APP can generate bioactive peptides other than β-amyloid(1–42) itself, including small C-terminal fragments that interact with signalling proteins (van der Kant and Goldstein 2015) and its intracellular domain (AICD50) retained after γ secretase cleavage which can act as a transcription factor (Konietzko 2012). Recent data demonstrate that AICD50 overexpression affects the levels of Sox2, a key transcription factor in the regulation of stem cell maintenance and lineage commitment (Sarlak et al. 2016). Since the physiological role of these cleavage products are still poorly understood, we focussed on β-amyloid as it has been correlated with cellular differentiation (Freude et al. 2011; Wang et al. 2009) and the peculiarity of accelerated ageing in Down syndrome (Obeid et al. 2016). We thus posit an increase of APP, and elevated levels of soluble β-amyloid (1–42) as a factor in promoting early brain maturation.

In our developmental Down syndrome cohort, we were not able to find β-amyloid plaques, suggesting that any pathological effect of β-amyloid is not due to its aggregation in foetal brains. Instead, we observed the first signs of plaque formation in tissues of a 15-year-old patient from a separate cohort, with more substantial deposits in patients > 30 years old. As β-amyloid oligomers are more toxic than un-aggregated peptide (Zhao et al. 2012), an increase in the soluble un-aggregated form could promote cellular morphological changes without compromising cell survival. In our foetal Down syndrome cases, we find elevated quantities of APP, and experimental challenges with external β-amyloid (1–42) delivery triggered the down-regulation of α3 subunits similarly to what is observed in human foetal brain. This observation is in line with our previous results, showing that developmental physiologic tau phosphorylation is disturbed in individuals with Down syndrome (Milenkovic et al. 2017), most probably by shifting the phosphorylation pattern towards an adult pattern. In sum, we attribute both APP and β-amyloid fragments as key features in the premature and erroneous differentiation of brain circuitry by deregulating α3 subunits, leading to miswired networks that suppress cognitive performance.

## Electronic supplementary material

Below is the link to the electronic supplementary material.

**Supplementary Fig.** **1** *Localization of GABA*_*A*_ *R subunits on cultured mouse primary neurons*. (**A-C**_**1**_) Cellular distribution pattern of GABA_A_R subunits on young neurons at 4DIV. Note the limited staining for α1 subunits at this age. *Arrowheads* and *open arrowheads* indicate presence and lack of staining, respectively. (**D-F**_**2**_) Cellular distribution pattern of GABA_A_R subunits at 7DIV when networks are forming. Note the opposition of subunits with VAMP2 and VGAT. *Arrowheads* point to IR while *open arrowheads* point to the absence of IR (n = nucleus). *Scalebars* = 30 µm (A,B,C,D,D_1_,E,E_1_,F,F_2_) (JPEG 1455 kb)

**Supplementary Fig.** **2** *GABA*_*A*_ *R* α*3 subunits localize to glial*-*like cells*. (**A-A**_**2**_) In *mouse* primary hippocampal cultures at 7 days of age, α3 IR can be found in the cytoplasm of large cells with glial-like morphology (TUJ1-negative with large nuclei), scattered within a neuronal network (TUJ1-positive). Note the punctate α3 staining resembling postsynaptic terminals (A_2_). *Scalebars* = 50 µm (A,A_1_) (JPEG 328 kb)

**Supplementary Fig.** **3** *GABA*_*A*_ *R subunits in the human fetal dentate gyrus*. (**A-C**_**1**_) Staining patterns of GABA_A_R subunits in the dentate gyrus of control cases. Arrowheads point to interneurons (A_1_,C). (**D,D**_**1**_) The α3 subunit is down-regulated in Down syndrome cases in the CA2/3 region of the hippocampus. (**E-H**_**1**_) IR quantifications of subunits in the inner and outer regions of the dentate gyrus (DGi and DGo, respectively). *p < 0.05. *Scalebars* = 100 µm (A,A_1_,C_1_,D,D_1_); 200 µm (B,C); 20 µm (B_1_) (JPEG 1423 kb)

**Supplementary Fig.** **4** *Beta amyloid effects on human SH*-*SY5Y neuroblastoma cells*. (**A-A**_**1**_) α3 subunit mRNA and protein are expressed in *mouse* (adult and p0), *human* hippocampus (Hu) and the *human* SH-SY5Y neuroblastoma cell line (SH). (**B-C**_**2**_) Micromolar concentrations of beta amyloid reduced cell viability. Abbreviations: Aβ, beta amyloid; Hu, human hippocampus; p0, neonatal; SH, SH-SY5Y neuroblastoma. *Scalebars* = 100 µm (C_2_) (JPEG 323 kb)

Supplementary material 5 (DOCX 22 kb)
