# Supplementary figures and images for: GABAA receptor subunit deregulation in the hippocampus of human foetuses with Down syndrome
Source: Brain Struct Funct. 2017 Nov 22;223(3):1501–18. doi: 10.1007/s00429-017-1563-3 (PMC5869939; doi:10.1007/s00429-017-1563-3)

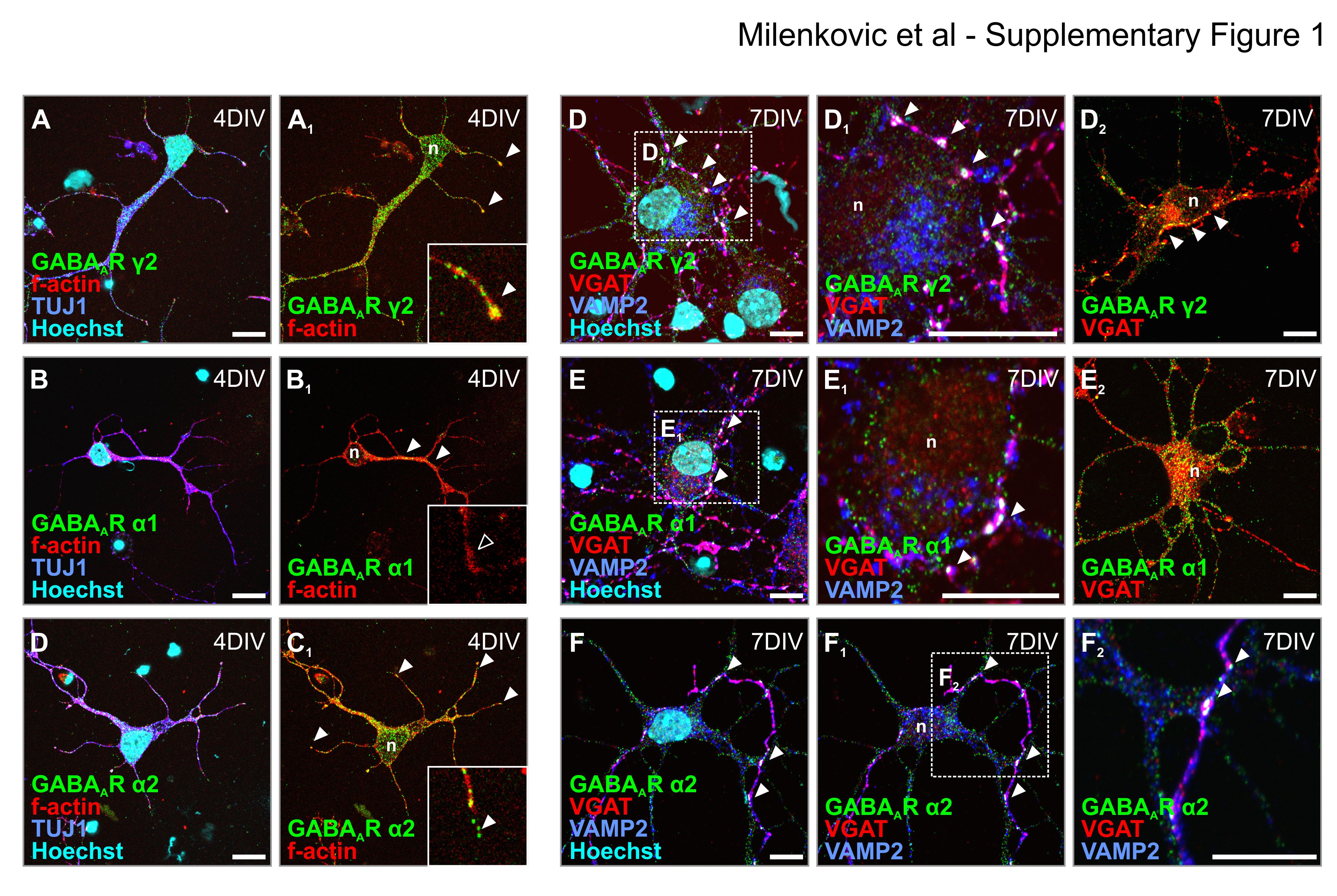

Supplement: Supplementary file 1 — Supplementary Fig. 1 Localization of GABA A R subunits on cultured mouse primary neurons. (A-C 1) Cellular distribution pattern of GABAAR subunits on young neurons at 4DIV. Note the limited staining for α1 subunits at this age. Arrowheads and open arrowheads indicate presence and lack of staining, respectively. (D-F 2) Cellular distribution pattern of GABAAR subunits at 7DIV when networks are forming. Note the opposition of subunits with VAMP2 and VGAT. Arrowheads point to IR while open arrowheads point to the absence of IR (n = nucleus). Scalebars = 30 µm (A,B,C,D,D1,E,E1,F,F2) (JPEG 1455 kb) [file 429_2017_1563_MOESM1_ESM.jpg]

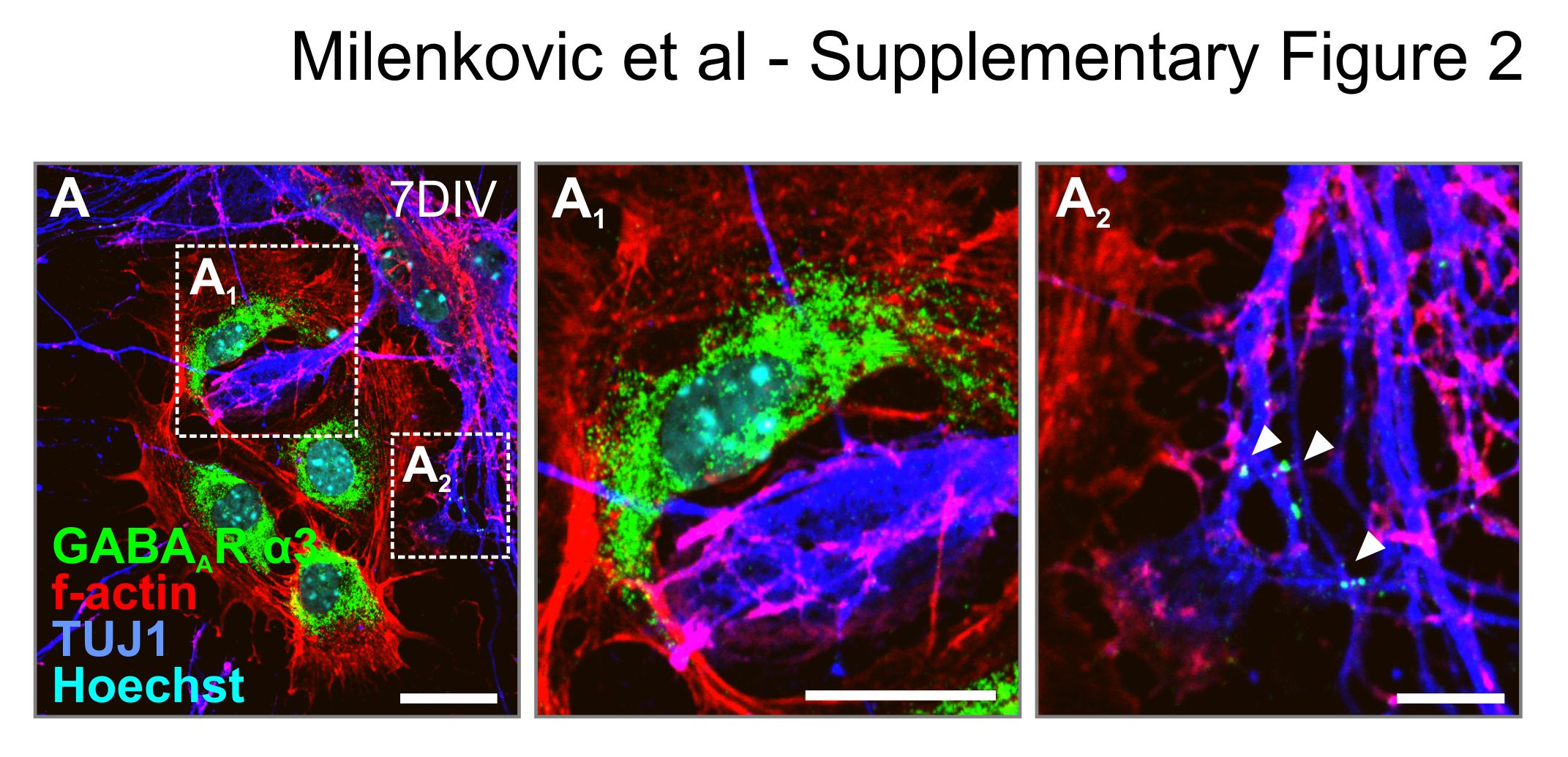

Supplement: Supplementary file 2 — Supplementary Fig. 2 GABA A R α3 subunits localize to glial-like cells. (A-A 2) In mouse primary hippocampal cultures at 7 days of age, α3 IR can be found in the cytoplasm of large cells with glial-like morphology (TUJ1-negative with large nuclei), scattered within a neuronal network (TUJ1-positive). Note the punctate α3 staining resembling postsynaptic terminals (A2). Scalebars = 50 µm (A,A1) (JPEG 328 kb) [file 429_2017_1563_MOESM2_ESM.jpg]

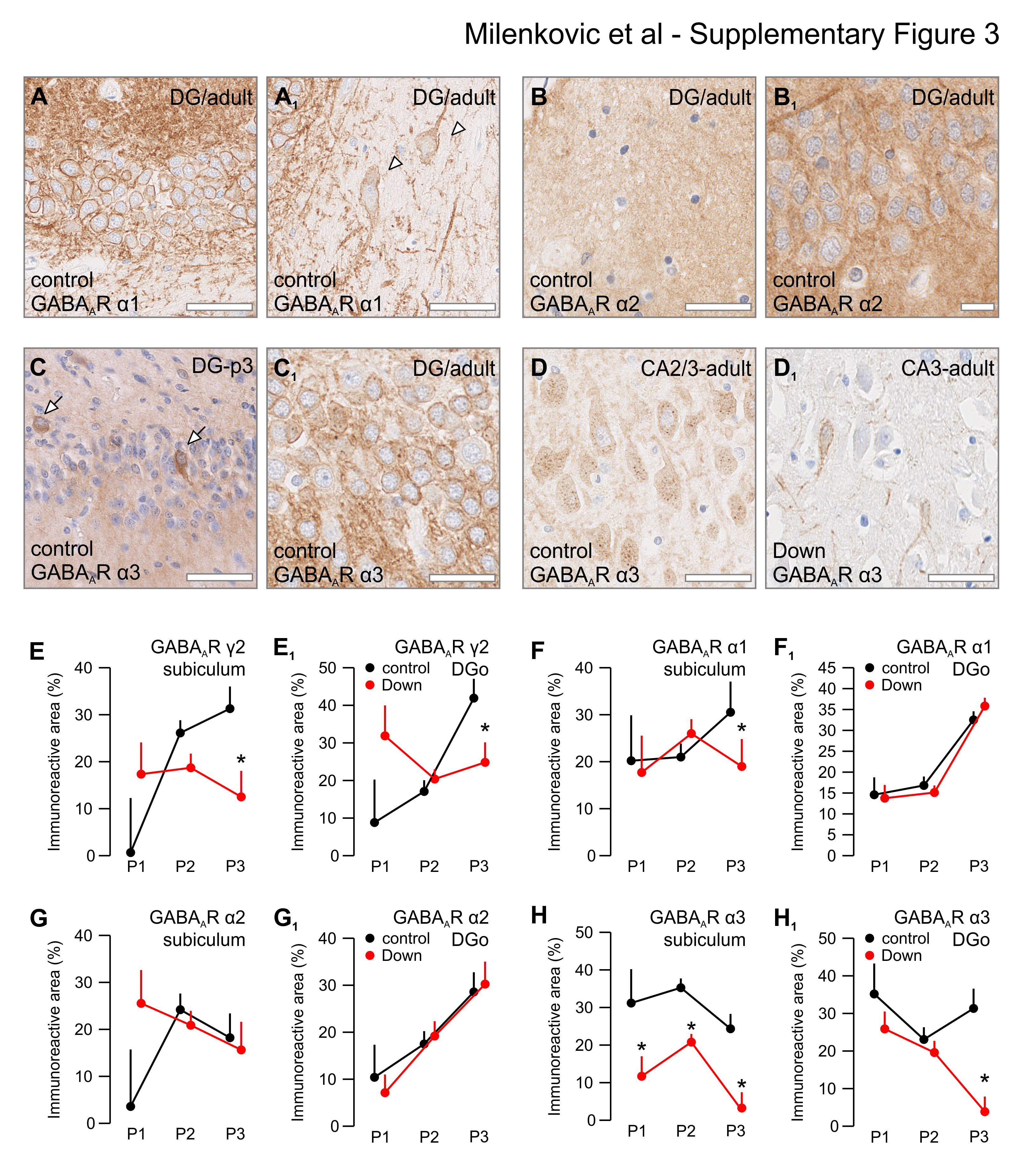

Supplement: Supplementary file 3 — Supplementary Fig. 3 GABA A R subunits in the human fetal dentate gyrus. (A-C 1) Staining patterns of GABAAR subunits in the dentate gyrus of control cases. Arrowheads point to interneurons (A1,C). (D,D 1) The α3 subunit is down-regulated in Down syndrome cases in the CA2/3 region of the hippocampus. (E-H 1) IR quantifications of subunits in the inner and outer regions of the dentate gyrus (DGi and DGo, respectively). *p < 0.05. Scalebars = 100 µm (A,A1,C1,D,D1); 200 µm (B,C); 20 µm (B1) (JPEG 1423 kb) [file 429_2017_1563_MOESM3_ESM.jpg]

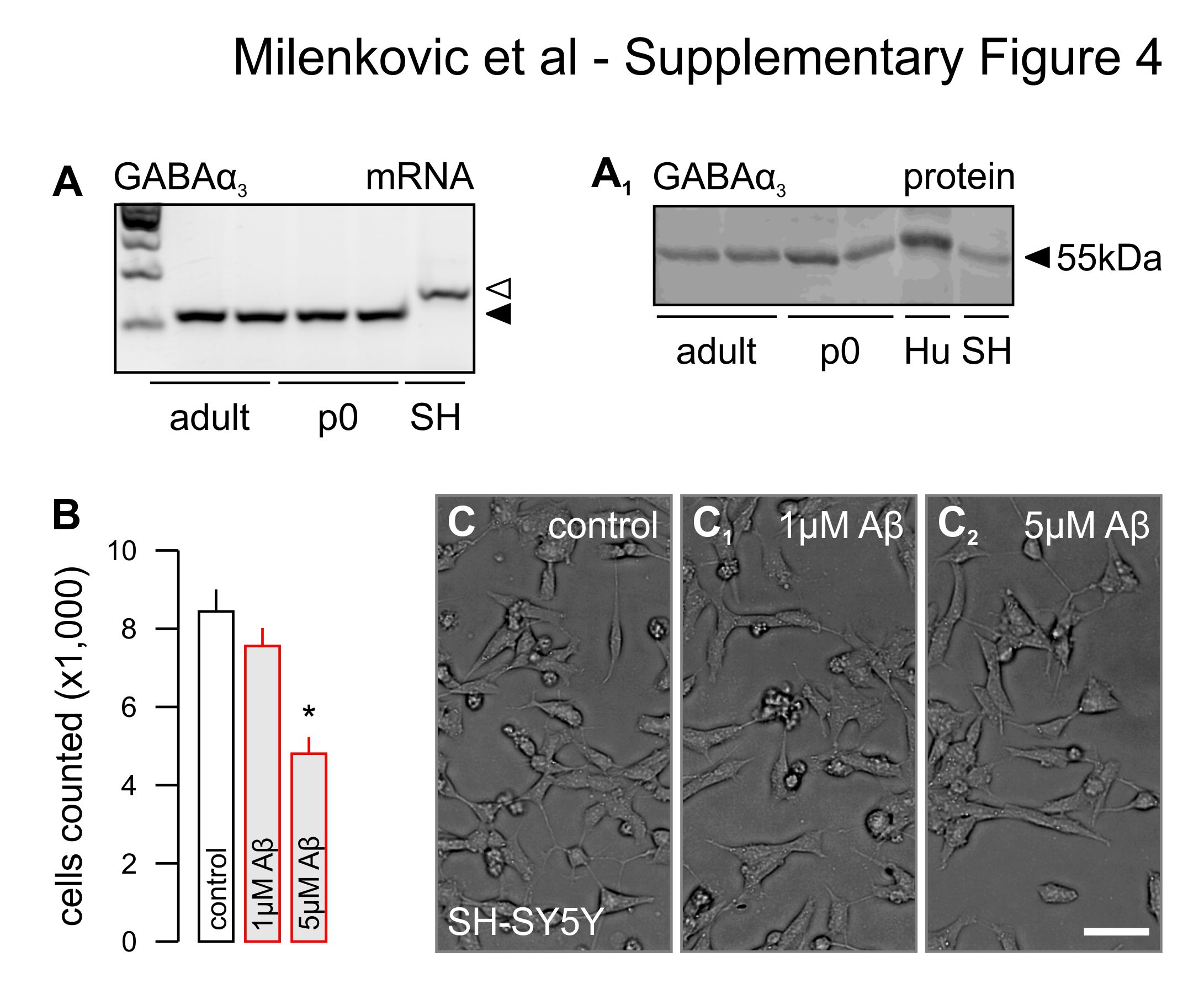

Supplement: Supplementary file 4 — Supplementary Fig. 4 Beta amyloid effects on human SH-SY5Y neuroblastoma cells. (A-A 1) α3 subunit mRNA and protein are expressed in mouse (adult and p0), human hippocampus (Hu) and the human SH-SY5Y neuroblastoma cell line (SH). (B-C 2) Micromolar concentrations of beta amyloid reduced cell viability. Abbreviations: Aβ, beta amyloid; Hu, human hippocampus; p0, neonatal; SH, SH-SY5Y neuroblastoma. Scalebars = 100 µm (C2) (JPEG 323 kb) [file 429_2017_1563_MOESM4_ESM.jpg]
