# Supplementary material for: GABAA receptor subunit deregulation in the hippocampus of human foetuses with Down syndrome
Source: Brain Struct Funct. 2017 Nov 22;223(3):1501–18. doi: 10.1007/s00429-017-1563-3 (PMC5869939; doi:10.1007/s00429-017-1563-3)
Supplement: Supplementary file 5 — Supplementary material 5 (DOCX 22 kb) [file 429_2017_1563_MOESM5_ESM.docx]

Supplementary Table 1 – List of cases used in this study. Abbreviations: BP, biological period; PL, postnatal life; GW, gestational week.

| Control | Gender | GW | PL | BP |  | Down | Gender | GW | PL | BP |
| --- | --- | --- | --- | --- | --- | --- | --- | --- | --- | --- |
| 1 | - | 15w3d | - | 1 |  | 1 | - | 15w4d | - | 1 |
| 2 | - | 17w | - | 2 |  | 2 | f | 15w4d | - | 1 |
| 3 | f | 17w6d | - | 2 |  | 3 | f | 15w5d | - | 1 |
| 4 | - | 19w | - | 2 |  | 4 | f | 15w6d | - | 1 |
| 5 | m | 19w3d | - | 2 |  | 5 | m | 16w | - | 2 |
| 6 | f | 19w4d | - | 2 |  | 6 | - | 16w | - | 2 |
| 7 | m | 19w4d | - | 2 |  | 7 | f | 16w4d | - | 2 |
| 8 | m | 20w6d | - | 2 |  | 8 | m | 18w | - | 2 |
| 9 | f | 21w1 | - | 2 |  | 9 | m | 18w | - | 2 |
| 10 | m | 21w2 | - | 2 |  | 10 | f | 18w4d | - | 2 |
| 11 | m | 22w | - | 2 |  | 11 | m | 18w5d | - | 2 |
| 12 | m | 22w4d | - | 2 |  | 12 | m | 19w5d | - | 2 |
| 13 | f | 23w | - | 2 |  | 13 | m | 20w | - | 2 |
| 14 | nn | 23w2d | - | 2 |  | 14 | m | 20w5d | - | 2 |
| 15 | m | 23w4d | - | 2 |  | 15 | f | 22w1d | - | 2 |
| 16 | m | 26w5d | - | 2 |  | 16 | m | 22w3d | - | 2 |
| 17 | m | 27w1d | - | 2 |  | 17 | m | 22w4d | - | 2 |
| 18 | m | 26w5d | 7d | 3 |  | 18 | f | 23w | - | 2 |
| 19 | f | 27w1d | 7d | 3 |  | 19 | m | 23w1d | - | 2 |
| 20 | f | 32w | 11d | 3 |  | 20 | f | 24w5d | - | 2 |
| 21 | f | 34w2d | 2d | 3 |  | 21 | m | 25w | - | 2 |
| 22 | m | - | 2.5mo | 3 |  | 22 | m | 22w1d | 1d | 3 |
| 23 | f | 32w5d | 4mo | 3 |  | 23 | m | - | 7d | 3 |
| 24 | - | - | - | adult |  | 24 | m | - | 11d | 3 |
|  |  |  |  |  |  | 25 | f | - | 5mo | 3 |
|  |  |  |  |  |  | 26 | f | - | 6mo | 3 |
|  |  |  |  |  |  | 27 | - | - | 15yrs | adult |
|  |  |  |  |  |  | 28 | - | - | 62yrs | adult |

Supplementary Table 2 – List of primer pairs used in this study.

| Probe name | Sequence (5’-3’) | Tm (˚C) | Size (bp) |
| --- | --- | --- | --- |
| GABAα_3_R Human Forward | CCG TCT GTT ATG CCT TTG TAT T | 56.5 | 165 |
| GABAα_3_R Human Reverse | TGT TGA AGG TAG TGC TGG TTT T | 56.5 |  |
| GABAα_3_R Mouse Forward | CTT GGG AAG GCA AGA AGG TA | 57.3 | 123 |
| GABAα_3_R Mouse Reverse | CCT TGG CCA GAT TGA TAG GA | 57.3 |  |

Supplementary Table 3 – List of antibodies used in this study. Abbreviations: Gp, guinea pig; Ms, mouse; Rb, rabbit.

| Marker | Immuno | WB | Host | Source |
| --- | --- | --- | --- | --- |
| APP | 1:100 |  | Ms | Chemicon |
| Brn-1 | 1:500 |  | Gt | Santa Cruz |
| DCX | 1:1,000 |  | Gp | Millipore |
| GABA_A_α1 | 1:300 |  | Rb | Dr. W. Sieghart |
| GABA_A_α1 | 1:300 |  | Ms | Millipore |
| GABA_A_α2 | 1:300 |  | Rb | Dr. W. Sieghart |
| GABA_A_α3 | 1:300 | 1:1,000 | Rb | Dr. W. Sieghart |
| GABA_A_γ2 | 1:300 |  | Rb | Dr. W. Sieghart |
| GFP | 1:1,000 |  | Gt | Abcam |
| Hoechst 33,342 | 1:10,000 |  | - | Sigma |
| Phalloidin-555 | 1:500 |  | - | Invitrogen |
| RC-2 | 1:100 |  | Ms | Millipore |
| SMI-32 | 1:2,000 |  | Ms | Dr. Sternberger |
| VAMP2 | 1:1,000 |  | Ms | Synaptic Systems |
| Vimentin | 1:200 |  | Ms | Dako |
| VGAT | 1:1,000 |  | Gp | Synaptic Systems |
| TUJ1 | 1:2,000 | 1:2,000 | Ms | Promega |
